# Macrophages employ quorum licensing to regulate collective activation

**DOI:** 10.1038/s41467-020-14547-y

**Published:** 2020-02-13

**Authors:** Joseph J. Muldoon, Yishan Chuang, Neda Bagheri, Joshua N. Leonard

**Affiliations:** 1 Interdisciplinary Biological Sciences Program, Northwestern University, Evanston, IL 60208 USA; 2 Department of Chemical and Biological Engineering, Northwestern University, Evanston, IL 60208 USA; 3 Center for Synthetic Biology, Northwestern University, Evanston, IL 60208 USA; 4 Chemistry of Life Processes Institute, Northwestern University, Evanston, IL 60208 USA; 5 Member, Robert H. Lurie Comprehensive Cancer Center, Northwestern University, Evanston, IL 60208 USA; 6 Biology and Chemical Engineering, University of Washington, Seattle, WA 98195 USA

**Keywords:** Tumour-necrosis factors, Monocytes and macrophages, Computer modelling, Differential equations

## Abstract

Macrophage-initiated inflammation is tightly regulated to eliminate threats such as infections while suppressing harmful immune activation. However, individual cells’ signaling responses to pro-inflammatory cues are heterogeneous, with subpopulations emerging with high or low activation states. Here, we use single-cell tracking and dynamical modeling to develop and validate a revised model for lipopolysaccharide (LPS)-induced macrophage activation that invokes a mechanism we term quorum licensing. The results show that bimodal phenotypic partitioning of macrophages is primed during the resting state, dependent on cumulative history of cell density, predicted by extrinsic noise in transcription factor expression, and independent of canonical LPS-induced intercellular feedback in the tumor necrosis factor (TNF) response. Our analysis shows how this density-dependent coupling produces a nonlinear effect on collective TNF production. We speculate that by linking macrophage density to activation, this mechanism could amplify local responses to threats and prevent false alarms.

## Abstract (web summary)

Macrophage activation is tightly regulated to maintain immune homeostasis, yet activation is also heterogeneous. Here, the authors show that macrophages coordinate activation by partitioning into two phenotypes that can nonlinearly amplify collective inflammatory cytokine production as a function of cell density.

## Introduction

In responding to external cues, cells are faced with many options, but by sharing information a population of cells can make more effective decisions than can each individual alone^1^. These decisions are generally mediated by secreted products. Bacteria use quorum sensing (QS) molecules to coordinate when and whether to form biofilms, and social amoeba secrete cyclic AMP to coordinate their aggregation. In each case, a proxy is used to convey information about the local number of cells available to coordinate the task.

In immunology, an established example of coordination is the amplification of the response to a perceived threat. During infection, cells such as macrophages provide an immediate line of defense by initiating inflammation to eliminate invading microbes^2^. This process often begins when the bacterial membrane component and pro-inflammatory cue lipopolysaccharide (LPS) is sensed by Toll-like receptor 4 (TLR4). Prior to TLR4 activation, the transcription factor NF-κB is sequestered in the cytoplasm by Inhibitor of κB (IκB). Upon activation, IκB kinase (IKK) targets IκB for degradation^3,4^, allowing NF-κB to translocate to the nucleus. There, NF-κB induces the transcription of genes such as tumor necrosis factor (TNF), a cytokine that mediates inflammation and pathogen clearance^5–7^. Other pathways downstream of TLR4 increase TNF production by stabilizing the mRNA and promoting translation and cleavage of the pro-protein for secretion. Extracellular TNF then signals through TNF Receptor 1 (TNFR1), driving NF-κB activation in positive feedback^8–12^, and this is a general explanation for how macrophages and other cells amplify their response to LPS.

The regulation of TNF has multiple layers. Recently, it was found that when the concentration of LPS exceeds a certain threshold, the induced signaling through NF-κB drives the transcription of its own RelA subunit in a process termed the feedback dominance (FBD) switch, producing *intra*cellular positive feedback on NF-κB expression and activity^13^. Other pathways act to constrain the response to LPS and ensure its eventual resolution: cell-intrinsic regulators (those with intracellular origins) include microRNAs and mRNA-binding proteins that decrease *Tnf* stability and translation^14^, as well as IκB^3,15^ and various inhibitors of IKK^4,8,16^ induced by NF-κB in negative feedback; cell-extrinsic regulators (those with extracellular origins) include interleukin 10 (IL-10), in that IL-10 signaling via the IL-10 receptor (IL-10R) antagonizes NF-κB activity and destabilizes *Tnf* stability and translation. In combination, these interlocking positive and negative motifs confer the functional plasticity necessary for immune cells to balance pathogen clearance with harmful side effects such as cytotoxicity and tissue damage^17^.

Given the many facets of the regulation of NF-κB and TNF, computational models have proven valuable for elucidating the properties of these systems and the roles of individual components. Early models explicated intracellular signaling^3,4,18–20^, and subsequent models included newly appreciated mechanisms such as intercellular feedback^8,10,11,21–24^. Recent studies have incorporated cell heterogeneity by attributing observed differences in gene expression either to stochastic fluctuations^25–27^ or to variation in initial values^28^, kinetic parameters^13,29–31^, or timing of signaling events^32^. A key consideration for understanding signaling and regulation in macrophages, in particular, is that these cells characteristically exhibit broad phenotypic heterogeneity^33–35^. It has been proposed that this variation could have important functional consequences, such as to broaden the repertoire of responses to stimuli^36^, propagate or restrain coordinated actions, or convert digital single-cell decisions into analog population-level ones^37^. While these ideas are interesting, specific mechanisms by which such heterogeneity might confer functional gain are not well understood.

In this study, we investigate the intriguing observation that when macrophages are treated with LPS, cell subpopulations emerge with high and low activation states. We propose a revised model in which macrophages use a process that we term quorum licensing to link the history of their density to the proportion of cells that become highly activated. This investigation provides new insights into how populations of macrophages use density information to regulate their collective activation.

## Results

### TNF expression is heterogeneous and varies with cell density

**Fig. 1:**
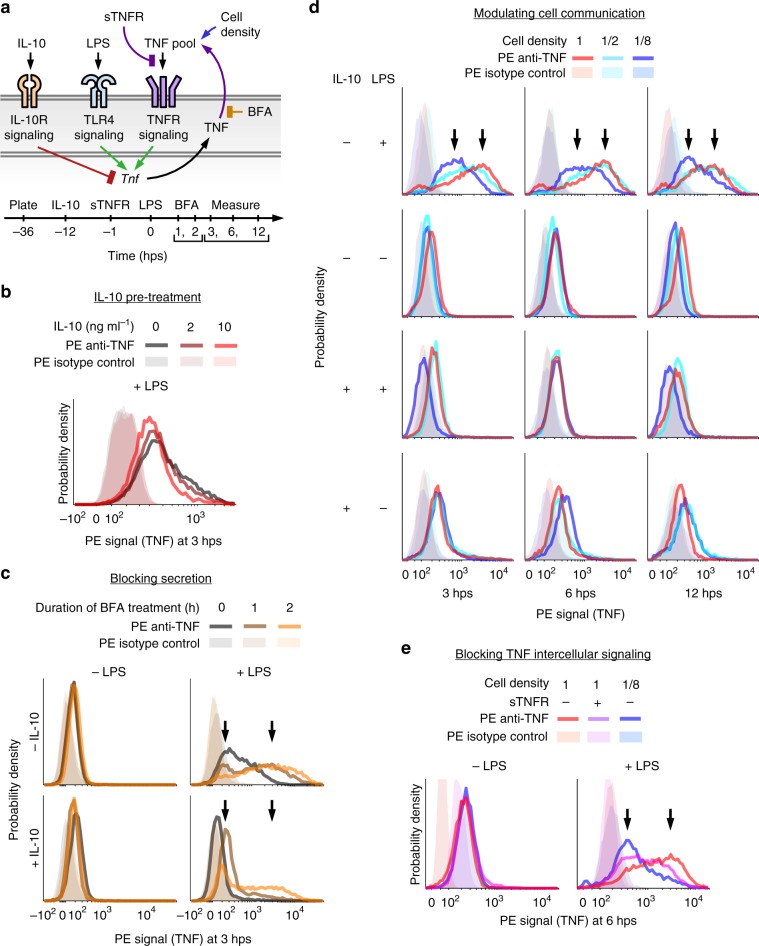
The TNF response to LPS is heterogeneous and requires intercellular communication. **a** The diagram summarizes the perturbations and stimuli applied to investigate TNF expression and intercellular communication (hps, hours post-stimulation with LPS). LPS activates TLR4 signaling, which induces TNF expression. IL-10 pretreatment activates IL-10R signaling, which inhibits LPS-induced TNF expression. Secreted TNF activates TNFR signaling, which induces TNF further through intercellular feedback. BFA prevents secretion, causing TNF to accumulate intracellularly. Varying the cell density modulates the concentrations of secreted factors such as TNF. sTNFR binds extracellular TNF and prevents TNFR signaling. **b** IL-10 pretreatment diminishes LPS-induced TNF expression. **c** TNF expression is heterogeneous with high-expressing and low-expressing subpopulations. After pretreatment with IL-10 (10 ng ml^–1^) and/or treatment with LPS (100 ng ml^–1^), cells were treated with BFA for 1 or 2 h. Arrows in **c**–**e** indicate low and high modes of the TNF distributions. **d** The full TNF response to LPS requires intercellular communication. **e** Intercellular feedback through secreted TNF is necessary for the full response. Source data are provided as a Source Data file.

Macrophage phenotypic heterogeneity has been observed in several studies^33–35^, and non-genetic heterogenous activation has been described in the widely used model cell line RAW 264.7^13,33^. We selected the RAW 264.7 model system to investigate how perturbations that modulate the response to LPS affect the heterogeneity with which macrophages become activated, as represented by expression of TNF (Fig. 1a). Pretreatment of cells with IL-10, prior to treatment with LPS, diminished the average intracellular TNF protein expression measured at 3 h post-stimulation (hps), although TNF distributions across IL-10 doses were broad and overlapping (Fig. 1b, Supplementary Fig. 1a). TNF expression was not highly correlated with flow cytometric proxies for cell size (Supplementary Fig. 1b), suggesting the heterogeneity was not due to cell cycle asynchrony alone.

To obtain a more direct readout of TNF production, we applied brefeldin A (BFA) to inhibit anterograde transport from the endoplasmic reticulum to the Golgi apparatus and prevent secretion. We reasoned that if variation in TNF secretion were a main source of heterogeneity, then BFA would diminish heterogeneity, and if gene regulation were the main source, then BFA would exaggerate it. Without LPS, BFA had no appreciable effect on intracellular TNF, indicating no detectable basal TNF production. However, when added after stimulation with LPS, BFA led to wide-ranging accumulation of on average several-fold more TNF cell^−1^ h^−1^ (Fig. 1c, Supplementary Fig. 1c). Unexpectedly, while most cells accumulated more TNF with longer BFA treatment, some cells accumulated little or no TNF over time. When cells were pretreated with IL-10 prior to LPS, TNF accumulation was less than when treated with LPS only, as expected, yet TNF accumulation was still wide-ranging for most cells and low for others. Therefore, blocking secretion unmasked substantial hidden variation in TNF production and showed that the cell population includes both high (wide-ranging) and low responders to LPS regardless of IL-10 pretreatment.

Since LPS-induced TNF intercellular signaling is known to contribute to NF-κB activity^8–12^, we examined the effect of intercellular communication on TNF production. To modulate communication in a manner that is not biased toward or against specific secreted factors, we varied cell density at plating (full, half, and one-eighth of previously used conditions) as a general handle for tuning the magnitude of coupling between cells. Without IL-10 and with LPS, a cell density-dependent effect was evident (Fig. 1d, Supplementary Fig. 1d). At 3 hps, average TNF expression correlated with density. At 6 hps, half and full density were TNF-high while one-eighth density remained low, and by 12 hps expression had decreased for each case. All of the distributions were heterogeneous, but at high density intracellular TNF remained skewed toward high expression over time and at low density it remained skewed toward low expression. With IL-10 or without LPS, little to no intracellular TNF was detectable. Thus, the full response to LPS requires intercellular communication, and cell density-associated effects on TNF production persist over time.

To investigate whether secreted TNF sustains its own LPS-induced expression, cells were pretreated with excess soluble TNF receptor (sTNFR) to titrate extracellular TNF from binding cell surface receptors. As TNF is bound by sTNFR, TNFR signaling is blocked^11^. With LPS, cells at high density with sTNFR pretreatment expressed TNF at an intermediate level on average—less than at high density without sTNFR, and more than at low density without sTNFR—and the distribution remained heterogeneous (Fig. 1e, Supplementary Fig. 1e). Therefore, although TNF intercellular feedback is required for full TNF production as expected, this mechanism does not explain the wide-ranging expression or the distinct high and low activation states observed. Thus, an additional explanation is required.

### The activation states’ proportions depend on cell density

To investigate the phenomena described above, we examined regulation upstream of the TNF protein using a previously validated clonal macrophage cell line with two genomically integrated reporters^13^ (Supplementary Fig. 2a). Such a reporter system enables one to resolve the dynamics and heterogeneity of individual cell signaling responses. The first reporter is a fusion of enhanced green fluorescent protein (EGFP) and RelA (the p65 subunit of NF-κB) driven by the *Rela* promoter. In the resting cell state, EGFP-RelA is sequestered primarily in the cytoplasm. Upon activation such as through TLR4 signaling, this protein translocates to the nucleus and induces the transcription of NF-κB target genes. Above a sufficient LPS dose, EGFP-RelA also induces the expression of endogenous RelA (and of EGFP-RelA) via an intracellular positive feedback loop termed FBD^13^. Thus, EGFP-RelA tracks both the localization and expression of NF-κB. The second reporter is a fusion of mCherry and a destabilizing PEST tag driven by the *Tnf* promoter. *mCherry* RNA lacks the *Tnf*-specific 3′ UTR and is decoupled from *Tnf*-specific post-transcriptional regulation. These features make the mCherry protein a proxy for transcription from the *Tnf* promoter (rather than for downstream TNF protein expression), after accounting for the time delay for mCherry translation and maturation^38^.

**Fig. 2:**
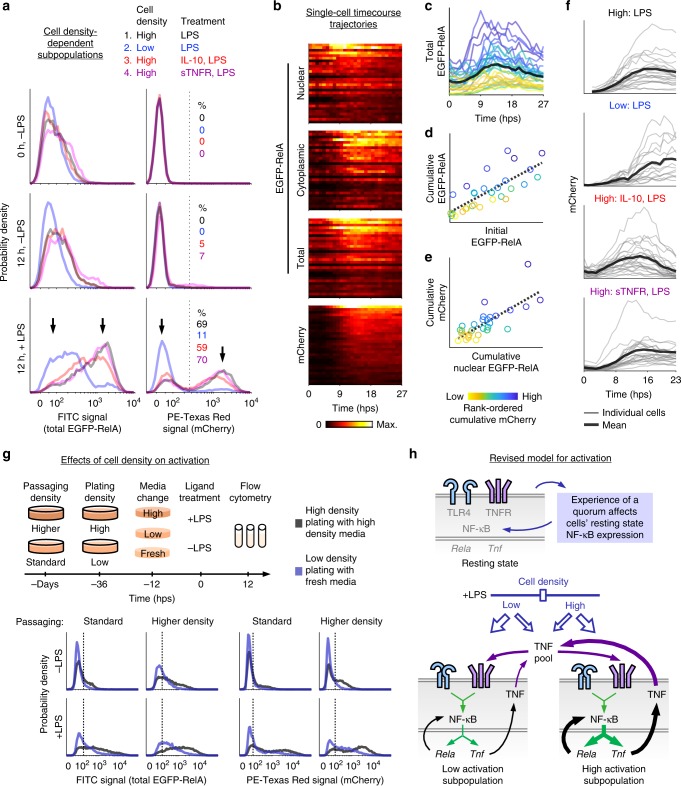
Cell density modulates the heterogeneity of macrophage activation. **a** Reporter protein fluorescence was measured by flow cytometry for the indicated cell densities, time points, and ligand treatments (IL-10 and sTNFR treatments as in Fig. 1). Percentages of highly activated cells were determined using a threshold (dotted vertical line) at the nadir between the two modes (arrows) of mCherry distributions. **b** Reporter trajectories for *n* = 30 cells at high density after treatment with sTNFR and LPS. Cells are ordered by cumulative mCherry expression and color-coded by fluorescence magnitude within heat maps. **c** Single-cell trajectories for total EGFP-RelA expression. The mean is in bold. **d**–**e** Relationship between initial and cumulative total EGFP-RelA (*R*^2^ = 0.61, one-tailed permutation test *p* = 2 × 10^−7^) and between cumulative nuclear EGFP-RelA and cumulative mCherry (*R*^2^ = 0.59, one-tailed permutation test *p* = 3 × 10^−7^). Dotted lines are linear fits, and axes are linearly scaled. In **c**–**e**, color-coding denotes rank-ordered cumulative mCherry expression. **f** Single-cell mCherry trajectories, with the mean in bold. Values are in a.u. specific to each panel. **g** Effect of culture density-associated conditions on macrophage activation heterogeneity. Fluorescence units are comparable within each reporter protein and passaging density. Dotted vertical lines distinguish low and high activation. **h** Revised conceptual model for macrophage activation with differently activated cell density-dependent subpopulations. Source data are provided as a Source Data file.

We utilized this reporter system to examine RelA expression and localization and *Tnf* promoter activity under the perturbations used above (Fig. 2a, Supplementary Fig. 2b). In all cases without LPS, EGFP-RelA and mCherry expression were low. With LPS, the mCherry distribution shifted from unimodal to bimodal, consistent with the observed expression of endogenous TNF (Fig. 1c). At the three high density conditions most cells were mCherry-high, and at low density a greater proportion was mCherry-low. The outcome at low density was not due to inhibition of EGFP-RelA nuclear translocation (Supplementary Fig. 2d). EGFP-RelA mirrored the pattern for mCherry, albeit with more overlap between modes, indicating an association between the induction of both reporters (Supplementary Fig. 2c). We also observed that average levels of TNF and mCherry ranked differently across conditions. In particular, cells at high density with IL-10 and LPS treatment had greater mCherry expression than did cells at low density with LPS treatment (Fig. 2a), yet this order was reversed for TNF expression (Fig. 1). The difference could be due to post-transcriptional downregulation of *Tnf* via IL-10R signaling^39–42^, which would diminish TNF protein more than *Tnf* transcription, and mCherry is a proxy for the latter. To investigate the possibility that the observed heterogeneity could be due to underlying variation in the reporter cell line, we generated monoclonal sublines from the parental reporter cell line (which was also generated monoclonally). An inducible bimodal phenotype was observed in each subline (Supplementary Fig. 2e–i), indicating that this phenomenon is a feature of the system and not due to clonal variation between cells.

Altogether, macrophage activation was bimodal, and subpopulation proportions varied with cell density. This observation held under perturbations to TNF-regulating pathways such as TNFR and IL-10R signaling. The dependence of the decision to become highly activated on cell density has some resemblance to bacterial QS, in that the phenotypes of individual cells are determined by information shared by the population. However, an important distinction is that in QS essentially all cells become activated if a threshold concentration of QS molecule is surpassed^43^, whereas here the *proportion* of highly activated cells increases with density. Therefore, because only a fraction of the cells become licensed to reach a state of high activation, the population exhibits an analog response rather than a digital one. To distinguish these two phenomena, we refer to the macrophage behavior as quorum licensing (QL).

### Single-cell analysis supports a revised model for activation

We next investigated whether heterogeneous activation was due to variation in the magnitude and/or timing of the response to LPS. Variation in magnitude would indicate a role for intrinsic or extrinsic noise, due to stochastic fluctuations or to deterministic outcomes of variation in initial (pre-LPS) conditions, respectively. Variation in timing could indicate a domino effect where early high-expressing cells activate other cells. To track the dynamics of EGFP-RelA expression and localization and mCherry expression, cells were stimulated and monitored over a one-day timecourse using confocal laser-scanning microscopy. Quantification of individual trajectories showed that *Tnf* promoter activation varied primarily in magnitude rather than timing (Fig. 2b). For EGFP-RelA (at high density with sTNFR and LPS), the mean expression increased and peaked at 14 ± 4 hps (± standard deviation among cells), after peak nuclear signal (10 ± 6 hps) and around peak cytoplasmic signal (15 ± 4 hps). The peak signal was greater in the cytoplasm than in the nucleus (1.4 ± 0.4 fold), and depletion of the nuclear portion mid-timecourse coincided with cytoplasmic accumulation. The expression of mCherry was initially low and then increased and peaked at 18 ± 5 hps. A small subpopulation of cells expressed very little mCherry or EGFP-RelA (consistent with flow cytometry observations for TNF and the reporters), and reporter expression appeared unrelated to which cells were in physical contact (Supplementary Fig. 2j). Intriguingly, although high induction of both reporters co-occurred in the same cells, the bimodality in TNF expression (measured at 3 hps in Fig. 1c) was evident *before* the observed increase in EGFP-RelA (Fig. 2b), even considering the ~1 h chromophore maturation time. This sequence of events was also observed for cells cultured at low density (Supplementary Fig. 2k). Since target gene expression would be expected to increase *after* an increase in the expression of a transcriptional regulator, the observed sequence indicates that FBD cannot be causal for the early TNF burst. Instead, these events are conditionally independent, i.e., regulated by an upstream process and not by each other.

The microscopy analysis revealed additional dynamical features of the single-cell responses. For nuclear-localized EGFP-RelA, while the average profile underwent an overall increase and subsequent decrease, some cells showed multiple peaks of varied amplitudes (Supplementary Fig. 2l, m) in agreement with a recent study^31^. There was also variation in an apparent nuclear reservoir of transcription factor (Fig. 2n, o), in agreement with another study^30^. For total EGFP-RelA, pre-LPS expression varied and was correlated with post-LPS cumulative expression (Fig. 2c, d, Supplementary Fig. 2p). The predictive power of this initial condition indicates a role for extrinsic noise (variation initially present in the system) in determining the post-FBD amount of transcription factor. Cumulative nuclear EGFP-RelA and cumulative mCherry were also correlated, as expected for a transcription factor and target gene (Fig. 2e). These outcomes indicate that extrinsic noise in RelA expression propagates to activity at the *Tnf* promoter. To further examine promoter activity, we quantified mCherry trajectories for each perturbation (Fig. 2f). At high density with LPS, mCherry increased for 16 ± 2 hps, indicating continued transcription after the intracellular TNF protein showed a decrease (Fig. 1d). This protein decrease is consistent with known post-transcriptional mechanisms that downregulate *Tnf*^14^, and such simultaneously opposing transcriptional and post-transcriptional regulation represents a type of control that has been compared to operating both the throttle and brake pedals of a vehicle^44^. Cells at low density took more time to reach peak mCherry signal compared to at high density. Cells pretreated with IL-10 increased in mCherry for 12 ± 3 hps and then decreased toward basal levels. Cells treated with sTNFR responded to LPS similarly to cells without this antagonist. Together, the measurements from flow cytometry and microscopy show how TNF is differently regulated under each perturbation (Supplementary Fig. 2q).

Another phenomenon related to cell density was that EGFP-RelA levels differed between low and high density both with and without LPS stimulation (Fig. 2a). This difference suggested that secreted factors might affect cells’ resting states in a way that predicts the response to LPS. To more carefully investigate how high density-associated conditions impact the patterns described above, we exposed cells to combinations of standard or higher density passaging (> 3 days), low or high density plating (at 36 h pre-LPS), and different conditioned media (fresh, low density, or high density; at 12 h pre-LPS). In general, exposure to more high density-associated conditions increased both the basal and induced reporter expression (Fig. 2g, Supplementary Note 1). For cells that were passaged at higher density and later provided conditioned medium from cells cultured at high density, the outcomes were similar regardless of the cell density at plating (which determines the number of cells present), indicating that sustained exposure to high density-associated secreted factors was sufficient to prime and enable full LPS-induced activation (Supplementary Fig. 2r). For the highest-density combination of conditions, reporter bimodality was prominent and right-shifted, even prior to LPS stimulation (Fig. 2g). The effect of passaging, which took place several days prior to plating at 36 h pre-LPS, indicates that the effects of culture density are heritable across cell generations. These observations motivate our proposal of a revised model for macrophage activation by QL, in which modifications to the extracellular milieu that occur during the resting state modulate the propensity for cells to become highly activated (Fig. 2h).

### A new computational model explains heterogeneous activation

**Fig. 3:**
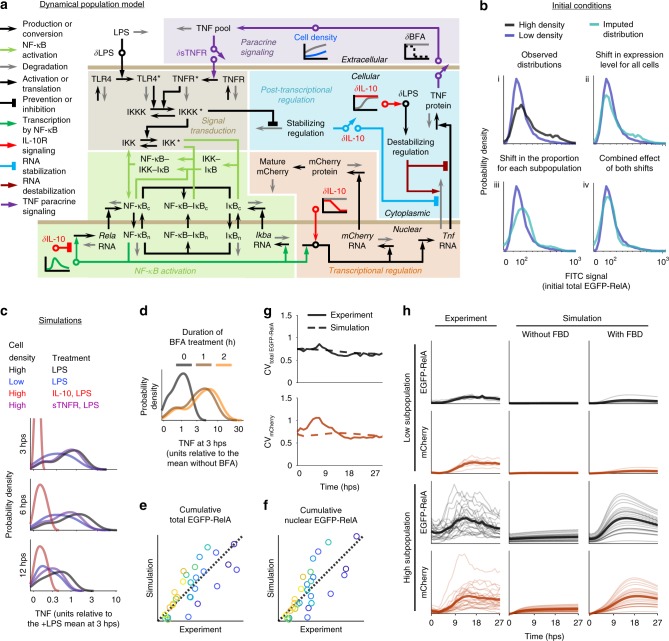
A dynamical population model explains intracellular and extracellular signaling and regulation. **a** The diagram summarizes variables, reactions, and mechanisms in the model of macrophage activation. Symbols: bold non-italicized text for variables; horizontal bars for compartment boundaries; n for nuclear and c for cytoplasmic; asterisks for activated receptors or kinases; delta symbol and circles for perturbation-specific effects; diagonal arrows for a perturbation’s effect; and graphs for time-dependent processes. **b** Generation of comparable distributions of initial values (data from Supplementary Fig. 2r). The observed distributions in (**i**) are in non-comparable units. To initiate simulations with NF-κB distributions that vary between high and low cell density and that match experimental observations for EGFP-RelA, we impute from an observed high density distribution (black) a low density distribution (teal) that matches the observed low density distribution (blue). The transform shifts the distribution (**ii**) and adjusts the proportion of cells in high vs. low states using a Gaussian mixture model of two populations fit to the high density distribution (**iii**), such that the combined transform (**iv**) generates an imputed distribution matching the observed low-density distribution (blue) with units relatable to high density (black). **c**–**d** Simulated TNF distributions from the calibrated model match experimental trends for cell density and ligand conditions in Fig. 1d, e and BFA conditions in Fig. 1c. All cells have the topology in **a** and are heterogeneous in initial value and transcription of *Rela* RNA and initial value of cytoplasmic NF-κB–IκB, derived from **b**. **e**–**h** Comparison of simulated and experimental outcomes for *n* = 30 cells at high density with sTNFR and LPS treatment. **e**–**f** Cumulative total EGFP-RelA (R^2^ = 0.61, one-tailed permutation test *p* = 3 × 10^−7^) and cumulative nuclear EGFP-RelA (R^2^ = 0.60, one-tailed permutation test *p* = 4 × 10^−7^), calculated using time-integrated values. The dotted diagonal is the identity line, and axes are linearly scaled. Color-coding is the same as in Fig. 2c–e. **g** Coefficient of variation (CV) in mCherry and total EGFP-RelA expression over time. **h** Trajectories were grouped post hoc by high or low activation. Mean values are in bold. Simulations are shown with and without FBD.

To integrate our observations with prior knowledge on macrophage activation, we developed a dynamical model for the intracellular and intercellular signaling network. We reasoned that such a framework could enable us to investigate how TNF is regulated and whether heterogeneity confers advantages to a population. Key aspects of model development were to include the most essential components, concisely portray biochemical processes, identify salient features of the data, fit parameters, and evaluate the extent to which simulations could explain the observations. To start, we examined studies on NF-κB and TNF^10–13,19,24,30,31,39–41,45–50^ and synthesized this information to produce a preliminary system of ordinary differential equations representing a cell that can inducibly express, secrete, and sense TNF. We reduced the model to decrease complexity^51^, and proposed, evaluated, and refined network topologies and their corresponding formulations (sets of equations) to produce the network depicted in Fig. 3a (Supplementary Methods).

Extrinsic noise featured prominently in the data. Therefore, we hypothesized that it might be possible to train a homogeneous model (a one-cell model) based on mean flow cytometry and microscopy data and subsequently to incorporate heterogeneity among cells. Parameters were split into two groups for separate rounds of fitting using parameter sweeps, multi-objective optimization, and an evolutionary algorithm (Supplementary Methods). The first round used a focused model of NF-κB activation with cell-intrinsic influences: TLR4 signaling; NF-κB activation, nucleocytoplasmic translocation, and inactivation; IκB expression; and FBD (Supplementary Fig. 3a, b). A fit to data for sTNFR and LPS (to exclude the influence of TNFR signaling) resulted in a family of parameter subsets with similar outcomes. The second round used the full model, incorporating *Tnf* transcriptional, post-transcriptional, and translational regulation; mCherry expression; effects of IL-10; TNF secretion and its inhibition by BFA; population growth (Supplementary Fig. 3c); and TNFR signaling. Each perturbation was simulated by modifying equations to capture its mode of action (Supplementary Fig. 3d). A simultaneous fit to all of the data, in which some parameters were constrained to subsets from the first round and others underwent a free search, yielded a suitable outcome (Supplementary Tables 1–4). We next adapted the model to represent a population coupled by TNF intercellular feedback, in which each cell varies in the basal state with extrinsic noise in NF-κB. Initial values for NF-κB were assigned based on EGFP-RelA confocal microscopy measurements (Fig. 2d). Additionally, we observed that EGFP-RelA distributions differed between high and low density by a shift in values and a shift between the two activation modes (Fig. 3b), and applying a transformation in silico to the initial values at high density could produce a distribution in comparable units for initial values at low density. In summary, the model was trained on homogeneous post-LPS data and initialized with heterogeneous pre-LPS data.

Remarkably, the use of varied pre-LPS EGFP-RelA levels, and the transformation of this distribution between high and low density conditions, enabled resulting simulations to capture the heterogeneity in the data, including intracellular TNF expression across perturbations and over time (Fig. 3c, compare to Fig. 1d, e) and TNF accumulation following treatment with BFA (Fig. 3d, compare to Fig. 1c). These simulations also accounted for the majority of the variation in cumulative transcription factor expression and localization (Fig. 3e, f). Furthermore, they closely tracked the distributions and trajectories of reporter expression (Fig. 3g, h) and supported the conclusion that both the high and low activation subpopulations underwent FBD (Fig. 3h). These findings support our strategy of first training the model on mean data and then introducing heterogeneity in a way that incorporates how the density-dependent pre-LPS state predicts the response to LPS.

### Model validated by observations from a distinct test dataset

**Fig. 4:**
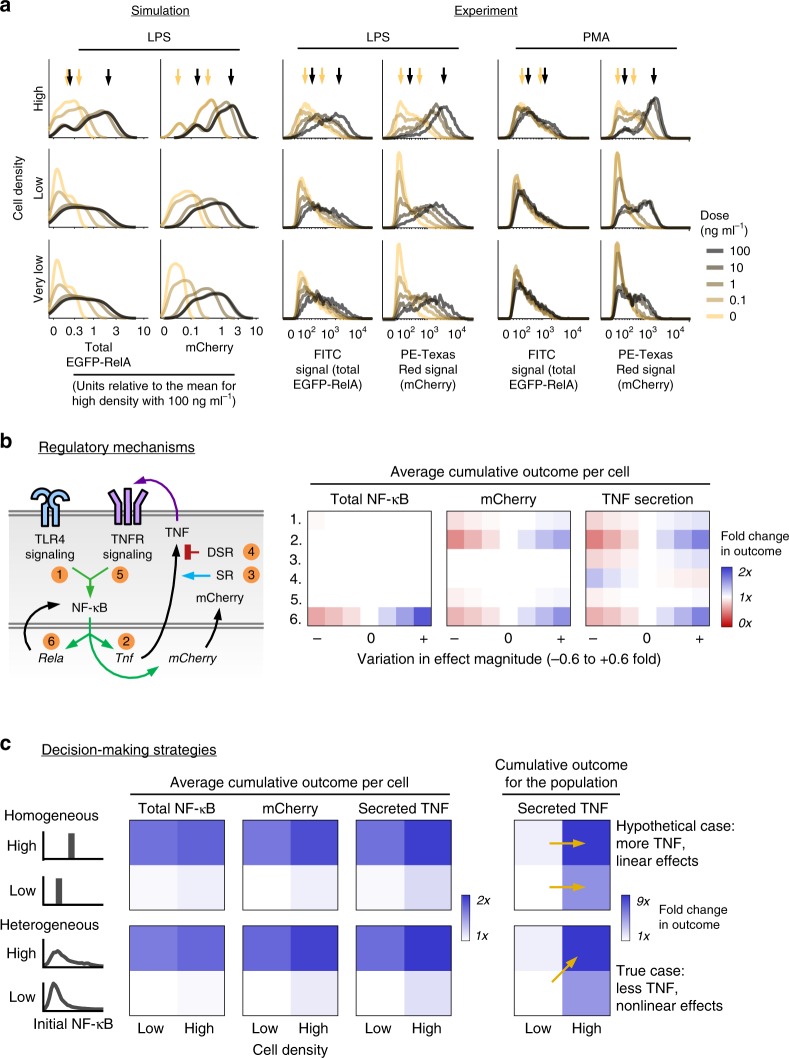
Mechanisms of TNF regulation differ in phenotypic and functional consequences. **a** Predicted and measured distributions of reporter expression across cell densities and doses of LPS or PMA at 12 hps. Very low density is 1/8th of low density. Arrows denote modes of the distributions for high density without stimulus or with 100 ng ml^−1^ of LPS or PMA. For simulations: low and very low density used the same initial values; mCherry distributions without stimulus were obtained without FBD and without intercellular feedback; and FBD was applied for LPS doses at and above 1 ng ml^−1^. **b** Simulated outcomes after individually varying the effect magnitudes of mechanisms that regulate TNF, with perturbations numbered and depicted in the diagram. Outcomes were assessed using the 12 hps time-integrated amounts of total EGFP-RelA, mCherry, and secreted TNF (cumulative secreted flux) per cell at high density. Conditions corresponding to the zero on the x-axis indicate base case effect magnitudes (*1×* on the color scale). Abbreviations: stabilizing regulation (SR) and destabilizing regulation (DSR). **c** Comparison of activation for homogeneous (hypothetical) or heterogeneous (observed) initial distributions of transcription factor expression. For each readout, a value of *1×* was set for the outcome given a heterogeneous population at low density with low initial values. Homogeneous initial values were set to the mean of corresponding heterogeneous distributions. In the right panel, the effect of the number of cells is incorporated into the total amount of secreted TNF. Arrows indicate comparisons from the main text, and key trends are noted on the right. Source data are provided as a Source Data file.

We sought to validate the model by testing whether it could predict responses to conditions not included in model development or fitting. To this end, we simulated populations at different densities and LPS doses. FBD was included for LPS treatment at or above 1 ng ml^−1^ (our estimate for a threshold at which this mechanism is active, based on the original study^13^). To obtain a test dataset, reporter cells were assayed under the same conditions (Fig. 4a, Supplementary Fig. 4a). Model predictions were broadly in agreement with the data: distributions for EGFP-RelA and mCherry had more rightward shifts with higher LPS doses, and two subpopulations were present with density-dependent proportions across doses. At lower doses and densities, the high-activation subpopulation showed diminished reporter expression, resulting in more overlap between subpopulations.

To test if any of the above observations were specific to TLR4 signaling, we evaluated the same conditions but for the stimulus used phorbol 12-myristate 13-acetate (PMA), a membrane-permeable small molecule that activates protein kinase C (PKC), which then activates IKK and by extension NF-κB. As was observed with LPS stimulation, PMA-induced expression of both reporters was bimodal with cell density dependence, and mCherry expression was ligand dose-dependent (Fig. 4a). Therefore, QL-associated activation of the *Tnf* promoter via NF-κB does not require that NF-κB be activated via TLR4. Interestingly, unlike trends observed with LPS, EGFP-RelA expression following PMA treatment differed little from the case without PMA, which indicates that FBD was induced by NF-κB activation via TLR4 but not via PKC.

### Simulations elucidate roles for TNF regulatory mechanisms

We next applied the validated model to examine roles of TNF regulatory mechanisms. First, we evaluated the robustness of simulations to global parameter variation by sampling parameter values from distributions with different coefficients of variation (CV) centered on fitted values. Variation was tolerated to some extent (Supplementary Fig. 4b). This tolerance is consistent with a general feature of systems biology models—sloppy parameter sensitivities^52^, in which various combinations of values yield similar outcomes. However, for variation beyond a CV of ~0.1–0.2 the outcomes began to diverge, suggesting sensitivity to certain mechanisms. We then used the model to explore how varying the effect magnitudes of six individual mechanisms (#1–6 in Fig. 4b, Supplementary Fig. 4c) would affect key readouts: the mean cumulative expression of NF-κB and mCherry (phenotypic consequences) and the total cumulative secretion of TNF (a functional consequence). The mCherry and TNF readouts were sensitive to transcriptional induction by NF-κB (#2), as expected. The TNF readout was the most sensitive to TLR4 signal transduction (#1), stabilizing regulation (#3), and destabilizing regulation (#4). Notably, FBD (#6) was the only mechanism to which the NF-κB readout was sensitive, indicating an apparent buffer from other processes, and all three readouts were affected by FBD, indicating FBD has wide-reaching effects. Also notable was that TNFR signal transduction (#5) affected the mCherry and TNF readouts only modestly, suggesting that even if cells were more tightly coupled by greater TNFR signal transduction, there would not be a large functional gain. Overall, the analysis showed that the most consequential mechanisms were FBD and transcriptional induction by NF-κB.

To investigate whether the observed heterogeneity could produce outcomes that differ from a hypothetical homogeneous population, we examined scenarios of high and low cell density in combination with high and low resting state levels of NF-κB. Homogeneous cells were assigned the mean value (without extrinsic noise) of NF-κB derived from the corresponding heterogeneous distribution, and LPS-induced outcomes were assessed by the three readouts. Unexpectedly, across scenarios, the homogeneous population was slightly more activated than was the heterogeneous one (Fig. 4c left panel, Supplementary Fig. 4d). Within both populations, initial conditions had a larger effect on the readouts for cells than did the number of cells (depicted by a greater distinction between rows than columns in the heatmaps). For total TNF secretion, both factors were important, and the coupling of initial NF-κB to cell density produced a greater fold change in TNF than would be the case if these factors were independent (Fig. 4c right panel, diagonal arrow vs. horizontal arrows). This coupling was also more consequential than the distinction between heterogeneity and homogeneity. Therefore, we conclude that it is not heterogeneity per se that is important, but rather that heterogeneity is regulated in a density-dependent manner; density controls the partitioning of a population of macrophages into high and low responders, yielding an effective strategy for driving population-level activation nonlinearly with cell density.

### Primary macrophages employ quorum licensing

**Fig. 5:**
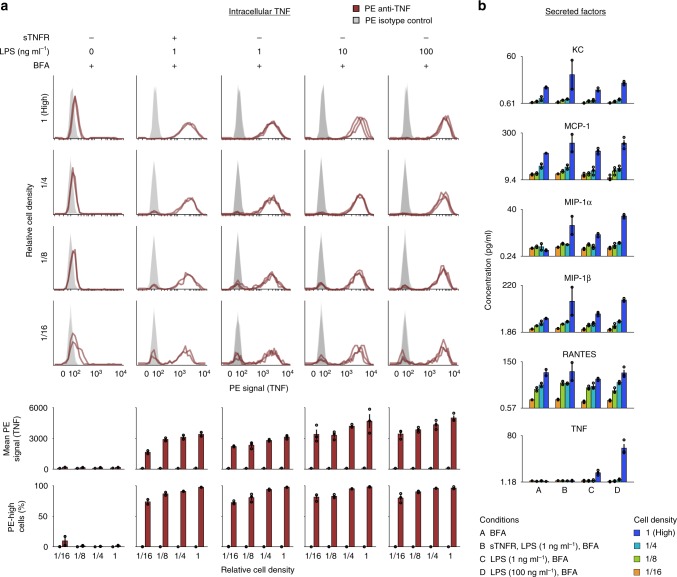
Primary macrophages regulate TNF production through quorum licensing. **a** Bone marrow-derived macrophages exhibit cell density-dependent bimodality in LPS-induced TNF expression. The first two conditions (columns) are in biological duplicate and the other three conditions are in biological triplicate. **b** Secreted factors were measured in cell culture supernatants. The minimum value on each *y*-axis is the observed lower limit of detection of the assay. A subset of the panel is included here, and the full panel is in Supplementary Fig. 5d–f. Bar graphs represent the mean of the biological replicates and S.E.M. Effects of cell density and treatment condition were assessed using two-factor ANOVAs and Tukey’s HSD tests (Supplementary Note 2). Source data are provided as a Source Data file.

To determine whether QL also occurs in primary cells, we generated murine bone marrow-derived macrophages (BMM) (Supplementary Fig. 5a–b). Cells were plated at different densities, subjected to combinations of stimuli, and assayed for intracellular TNF expression at 3 hps. In each of two independent experiments, we observed that BMM exhibit the key features of QL: LPS-induced TNF expression was bimodal; the proportion of cells in the high-activation mode increased with cell density; and TNF expression was bimodal even when cells were pretreated with sTNFR or IL-10 (Fig. 5a, Supplementary Fig. 5c). This outcome is consistent with observations in RAW cells and reporter cells (Figs. 1–2) that QL is independent of canonical TNF-regulating pathways including LPS-induced TNF intercellular feedback.

Lastly, to investigate how cell density might impact the products secreted by BMM, we used a multiplexed assay to evaluate cytokines and chemokines in the supernatant (Fig. 5b, Supplementary Fig. 5d–f). At high cell density, there was a large boost in secreted TNF. This nonlinear scaling of collective TNF secretion with cell density supports our proposed explanation that this is a central functional consequence of QL. Additionally, the assay ruled out several analytes—those that were not basally secreted and thus cannot coordinate the population to be poised for activation, and those that did not trend with density—as candidate mediators of QL. Of the twenty-three analytes evaluated, only the five chemokines examined (KC, MCP-1, MIP-1α, MIP-1β, and RANTES) were detected in the basal state and generally increased with cell density. Although these factors exhibit several necessary features of a QL mediator, chemokine receptor signaling activates multiple intracellular pathways, and evaluation of causality requires substantial further investigation (Discussion). Nonetheless, these observations demonstrate that QL occurs in primary macrophages, mediating a nonlinear relationship between cell density and population-level TNF production.

## Discussion

This study explores the observation that within a population of genetically identical macrophages, a potent activating stimulus (LPS) drives high expression of TNF in only a subset of cells. The observation of TNF heterogeneity is consistent with a recent study that showed cellular responses vary widely in response to such cues^53^. In examining the regulation that underlies this variation, we observed heterogeneity in transcription factor expression and localization, *Tnf* promoter activation, and TNF expression. Furthermore, the measured distributions showed states for high (wide-ranging) and low activation. This bimodality was tunable by modulating conditions related to cell density, indicating a role for intercellular communication potentially via secreted proteins, metabolites, or extracellular vesicles^54^. Using single-cell tracking, dynamical modeling, and primary cell experiments, we proposed, developed, and validated a revised model for activation by QL, in which a population’s experience of density over time impacts the extent to which cells become poised for activation.

Although bacterial QS provides a useful conceptual reference point, QL more closely resembles other types of phenotypic bimodality, which have been observed in native and synthetic contexts, across species, and stem from various mechanisms. Some of these mechanisms do not require intercellular communication. For example, continuous variation in protein expression in a population can produce digital outcomes for kinase activity in cells due to activation thresholds^55^, and intracellular positive feedback on synthetic promoters can support stochastic activation through transcriptional bursting^56^. Other instances of phenotypic bimodality do use intercellular communication. For example, in T cells, secretion of the cytokine IL-2 feeds back through IL-2R signaling and promotes further IL-2 expression. As the sensing involves capturing extracellular IL-2, feedback is confined locally and only some cells sense high amounts;^57^ TNF intercellular signaling similarly involves competitive uptake of TNF^58^. In the case of a synthetic genetic circuit in yeast with topological similarity to TNF intercellular signaling, two modes of activation were observed with proportions that varied with cell density^59^. Mathematical modeling analysis revealed that if cells rely on autocrine signaling more at low density than at high density, and if at low density few cells activate through autocrine signaling alone, then at high density a greater proportion of cells should activate as the balance shifts toward paracrine signaling via a shared cue. QL could belong to this same family of bimodal activation behaviors. It could even comprise a broader phenomenon—many genes are regulated by pathways that overlap with those that regulate expression of TNF^41^, and single-cell RNA-seq analyses have revealed bimodality in hundreds of immune response genes^60,61^. In addition, macrophages were recently found to use a quorum-based mechanism to resolve inflammatory processes via production of nitric oxide^62^. Investigating the various mechanisms by which heterogeneity and coordination interact represents an exciting avenue for future investigation.

Our study suggests nuanced interpretations for several phenomena. First, it was recently shown that NF-κB can induce RelA in intracellular positive feedback termed the FBD switch^13^. We found that this effect persists over the long-term and coincides with sustained *Tnf* promoter activity. However, these dynamics differ from those of intracellular TNF protein, which undergoes a burst (in which bimodality in its production is evident) before FBD takes effect, and then begins to decline before mCherry reaches peak expression. Second, phenotypic variation among genetically identical cells is often attributed to stochasticity (intrinsic noise). However, in this system, the predictive power of the pre-LPS state indicates a substantial role for extrinsic noise. If additional species were measured to more fully characterize the pre-LPS state, we anticipate that the multivariate initial conditions would provide further predictive power as to how individual cells are poised to respond to LPS. Third, we observed an inherited density-dependent propensity for activation involving an accumulation of transcription factor in the resting state. This result suggests that one way in which a population can become and remain at least temporarily poised for high activation is for cells to stockpile a transcription factor (or conceivably other molecules), and in the absence of continued high density-associated conditions, these levels would eventually decrease. Whatever the underlying mechanisms are for maintaining long-term activation propensity, a strategy that enables cells to calibrate their response to future threats by integrating their experience of the surroundings over time (including information about whether other cells have been recruited to that site) could provide functional utility.

A fourth insight is that even though the role of TNF in intercellular communication is established^8–12^, we found that as an intercellular signal, TNF had a modest influence on FBD and *Tnf* promoter activity. Intercellular TNF signaling had a larger effect on TNF protein expression, consistent with known mechanisms through which TNFR signaling enhances TNF expression through post-transcriptional regulation^48^. Since chronic TNF production is implicated in various diseases, therapeutic strategies have focused on blocking TNF from binding surface receptors by administering anti-TNF antibodies or sTNFR^63^ or by administering antagonists of TLR4 or associated proteins^64^. While we did not attribute QL to specific mediators, if these factors can be identified in future work, they could represent targets for immunomodulation. For example, the BMM secretion assay showed that chemokines accumulate with cell density prior to LPS treatment—a necessary feature of any candidate mediator. It has been shown that chemokines mediate leukocyte recruitment to sites of infection^65–68^, chemokine receptor signaling regulates NF-κB activity^69–71^, and NF-κB itself induces the expression of certain chemokines^72^. While the observations from the secretion assay are intriguing, there are many chemokines and they signal through overlapping pathways, and thus our findings identify an opportunity for future investigation as to whether these molecules have a role in QL.

Looking beyond the scope of this investigation, it is interesting to speculate whether QL may be adaptive for immune function. Such a mechanism could provide a way for a cell population to preemptively coordinate a response to microbial incursion prior to LPS-induced intercellular signaling. As an example, we consider a scenario in which a wound is experienced, resulting in microbial incursion. Since tissue damage can immediately trigger local sterile inflammation (via damage-associated molecular patterns, DAMPs)^73^, macrophages among other cells could locally accumulate and become primed for high activation through QL. Then, should replication of invading microbes produce more pathogen-associated molecular patterns (PAMPs) such as LPS, the local inflammatory response would escalate. Another consequence would be to limit potent inflammation to local environments. In the scenario posed above—a wound experiencing an infection—some DAMPs could travel to sites that are remote from the wound, even if the microbes are limited to the wound site. QL could act to limit the most potent macrophage-mediated responses to sites characterized by *both* the presence of DAMPs and the local accumulation of macrophages, nonlinearly driving local cytokine production. If macrophage recruitment could be coupled to activation in this way, then QL would enhance the specificity of this potent but potentially harmful facet of innate immune function. Conversely however, for conditions such as sarcoidosis, fibrosis, and atherosclerosis^74,75^ characterized by abnormal accumulation of macrophages and related cells, QL would be maladaptive by supporting chronic inflammation. These ideas each comprise compelling avenues for future investigation, building upon the insights gained here into mechanisms linking heterogenous immune cell activation to intercellular communication.

## Methods

### RAW cell culture

RAW 264.7 cells were cultured in complete DMEM medium containing 1% DMEM (Gibco #31600091), 10% heat-inactivated FBS (Gibco #16140071), 4 mM L-glutamine (Gibco #25030081), and 100 U ml^−1^ penicillin and 100 μg ml^−1^ streptomycin (Gibco #15140122) in tissue culture-treated dishes (Corning) at 37 °C in 5% CO_2_. To passage, medium was aspirated, and cells were washed in PBS, incubated in PBS-EDTA (5 mM EDTA in PBS pH 7.4) (37 °C, 5 min), detached by gentle scraping, pelleted by centrifugation in 50 ml conical tubes (125 × *g*, 5 min), and resuspended in fresh medium and plated. Reporter cells were cultured under the same conditions. For higher density passaging (Fig. 2g, Supplementary Fig. 2r), reporter cells were grown to cover a large majority of surface area of dishes, and this condition was maintained for several days leading up to the functional experiment. RAW 264.7 cells were a gift from David Segal (NIH), and reporter cells^13^ were a gift from Iain Fraser (NIH). Cell lines were not authenticated further.

### Confocal microscopy

Experiments were conducted using reporter cells and a Zeiss inverted Axio Observer Z1 confocal microscope with a custom environmental control chamber for CO_2_, humidity, and temperature control. Data were exported, and fluorescence was quantified using ImageJ software^76^: at each time point, the mean signal for each cell was quantified and the mean background signal based upon multiple sampled regions without cells was subtracted. The resulting value was multiplied by the area enclosed by the plasma membrane in the image slice to determine EGFP-RelA signal and mCherry signal. A similar method was applied using the area within the nuclear membrane to determine nuclear EGFP-RelA signal. For conditions at high density, 30 cells were quantified; cells were not quantified if they divided or exited the field of view during the timecourse. For low density, 20 cells were quantified; due to the low number of trackable cells within the field of view in this case, some traces start or end within the timecourse.

### Generation of monoclonal lines

The original reporter cell line was generated^13^ via lentiviral integration of the two reporters encoded in a single vector—using a low multiplicity of infection, such that the large majority of cells underwent at most one integration^77^—followed by fluorescence-activated cell sorting for EGFP-positive clones, clonal expansion, and functional screening for a clone exhibiting LPS-inducible EGFP-RelA nuclear translocation and mCherry expression. In the current study, seven monoclonal sublines (labeled A–G) were generated from the parental line by limiting dilution and clonal expansion.

### L929 cell culture

L929 fibroblasts (ATCC) were cultured in RPMI-1640 medium (Gibco #11875093, containing L-glutamine) supplemented with 10% heat-inactivated FBS, 100 U ml^–1^ penicillin, and 100 μg ml^–1^ streptomycin in tissue culture-treated dishes at 37 °C in 5% CO_2_. For passaging, medium was aspirated, and cells were washed in PBS, incubated in trypsin-EDTA (Gibco #25300054) (37 °C, 5 min), detached by tapping the dish and pipetting in fresh medium, pelleted by centrifugation in 50 ml conical tubes (150 × *g*, 5 min), resuspended in fresh medium, and plated. L929-conditioned medium was prepared by filtering supernatant at 2 or 3 days after the previous passage, by which time the cells had covered a large majority of the surface area of dishes. Conditioned medium was stored at –20 °C and thawed for differentiating primary cells.

### Bone marrow harvest and primary cell culture

C57BL/6 male mice (Jackson Labs; 5–10 weeks old) were sacrificed, and bone marrow cells were harvested from femurs and tibias^78^. All of the animals were handled according to the animal protocol (#IS00003438), which was approved by the Northwestern University Institutional Animal Care and Use Committee (IACUC) and complies with all relevant ethical regulations for animal testing and research. Cells were cultured in differentiation medium containing complete RPMI-1640 medium (as used for culturing L929 cells) supplemented with 10% L929-conditioned medium in non-treated 10 cm dishes (Corning #CLS430591) (4 × 10^4^ monocytic cells ml^–1^, 10 ml per dish) at 37 °C in 5% CO_2_. At 3 days post-harvest, medium was aspirated without removing the partly adherent cells and replaced with differentiation medium containing fresh complete RPMI-1640 medium supplemented with 15% L929-conditioned medium. Cells were cultured for four more days, by which time the cells had become adherent BMM.

### BMM surface staining

At 7 days post-harvest, BMM were stained for surface markers of differentiation and assayed by flow cytometry. Medium was aspirated, and cells were washed in PBS, incubated in FACS buffer (FB; 5 mM EDTA and 0.1% BSA in PBS) (4 °C, >10 min), detached by tapping dishes firmly and pipetting in FB, aliquoted as 2.5 × 10^5^ cells per FACS tube, and pelleted by centrifugation (400 × *g*, 5 min). Supernatant was decanted, paraformaldehyde (PFA; 4% in PBS, 30 μl) was added, and tubes were flicked to mix and incubated (4 °C, 20 min). 1 ml FB was added, tubes were flicked to mix and centrifuged, and supernatant was decanted; this step was performed a total of three times. To block, normal mouse serum (Sigma #M5905, 10 μl) was added, and tubes were flicked to mix and incubated (room temperature—approximately 22 °C, 15 min). Staining was conducted with primary conjugated antibodies (BD Biosciences): PE rat anti-CD11b (#553311, 0.04 μg) and Alexa Fluor 647 rat anti-mouse F4/80 (#565854, 0.04 μg). Isotype controls were PE rat IgG2b, κ isotype control (#553989, 0.04 μg) and Alexa Fluor 647 rat IgG2a, κ isotype control (#557690, 0.04 μg). Compensation control samples were prepared using anti-surface marker antibodies separately, and a no-antibody control sample was prepared. Tubes were flicked to mix and incubated (4 °C, 1 h). 1 ml FB was added, tubes were flicked to mix and centrifuged, and supernatant was decanted; this step was performed a total of three times. Several drops of FB were added, and tubes were covered in foil and kept on ice until flow cytometry.

### RAW functional experiments

High density (3.3 × 10^5^ cells ml^–1^) and low density (4.1 × 10^4^ cells ml^–1^) conditions used 1.35 ml of cell culture per well of a 6-well plate. The very low density condition for reporter cells (5.2 × 10^3^ cells ml^–1^) used 8 ml of cell culture in a 10 cm dish. Ligand treatments included recombinant mouse IL-10 (R&D Systems #417-ML, 10 ng ml^–1^ except as indicated in Fig. 1b) at –12 hps, recombinant mouse sTNFR (R&D Systems #763208, 8.3 μg ml^–1^) at –1 hps, *E. coli* 055:B5 LPS (Sigma-Aldrich, varied doses) or PMA (Cayman Chemical #10008014, varied doses) at 0 hps (36 h post-plating), and BFA (2 μg ml^–1^, Sigma-Aldrich #B5936) at 1 or 2 hps. Medium was not exchanged during ligand treatments. Cells were harvested at the time indicated for each experiment (3, 6, or 12 hps).

### BMM functional experiments

At 7 days post-harvest, medium was aspirated, and cells were washed with PBS, incubated in PBS-EDTA (>10 min), and detached by firmly tapping plates and pipetting in PBS-EDTA. Cells were centrifuged in 50 ml conical tubes (400 × *g*, 5 min), supernatant was discarded, and cells were resuspended in complete RPMI-1640 medium without L929-conditioned medium and replated at varying densities: high density in non-treated 6-well plates (Falcon #351146) (3.3 × 10^5^ cells ml^–1^, 1.35 ml per well) and 1/4th, 1/8th, and 1/16th of high density in non-treated 10 cm dishes (8 ml). Ligand treatments included IL-10 (10 ng ml^–1^) at –12 hps, sTNFR (8.3 μg ml^–1^) at –1 hps, LPS (varied doses) at 0 hps, and BFA (2 μg ml^–1^) at 1 hps. Medium was not exchanged during ligand treatments. Cells were harvested at 3 hps. Conditions were carried out in biological replicates indicated in figure legends.

### TNF staining for RAW cells and BMM

Medium was aspirated, and cells were washed with PBS, incubated in FB (37 °C, 5 min for RAW cells; 4 °C, >10 min for BMM), detached from plates (by gentle scraping for RAW cells; by pipetting for BMM), pipetted into two FACS tubes per sample (one for the anti-TNF stain and one for the isotype control stain), and pelleted by centrifugation (150 × *g*, 5 min for RAW cells; 400 × *g*, 5 min for BMM). Supernatant was decanted, PFA was added, and tubes were flicked to mix and incubated (4 °C, 20 min). 1 ml FB was added, tubes were flicked to mix and centrifuged, and supernatant was decanted; this step was performed a total of three times. 1 ml permeabilization wash buffer (PWB; 0.5% saponin and 0.1% BSA in PBS) was added, tubes were flicked to mix and centrifuged, and supernatant was decanted; this step was performed a total of two times. To block, normal mouse serum (10 μl) was added, and tubes were flicked to mix and incubated (room temperature, 20 min). PE-conjugated rat anti-mouse TNF antibody (BD Bioscience #554419, 0.1 μg) or PE-conjugated rat isotype control antibody (BD Bioscience #554685, 0.1 μg) was added, and tubes were flicked to mix and incubated (4 °C, 1 h). 1 ml PWB was added, tubes were flicked to mix and centrifuged, and supernatant was decanted; this step was performed a total of three times. Several drops of FB were added, and tubes were covered in foil and kept on ice until flow cytometry.

### Preparation of reporter cells for flow cytometry

Medium was aspirated, and reporter cells were washed with PBS, incubated in FB (37 °C, 5 min), detached from plates by gentle scraping, pipetted into FACS tubes, and pelleted by centrifugation (150 × *g*, 5 min). Cells were fixed to prevent loss of reporter signal: supernatant was decanted, PFA was added, and tubes were flicked to mix and incubated (4 °C, 20 min). 1 ml FB was added, tubes were flicked to mix and centrifuged, and supernatant was decanted; this step was performed a total of three times. Several drops of FB were added, and tubes were covered in foil and kept on ice until flow cytometry.

### Flow cytometry

Samples were run on a BD LSRII or BD LSR Fortessa flow cytometer using the FITC channel for EGFP-RelA, PE-Texas Red channel for mCherry, PE channel for TNF intracellular staining and for CD11b surface staining, and APC channel for F4/80 surface staining. Data were gated using FlowJo software on live (FSC-A vs. SSC-A) and single-cell (FSC-A vs. FSC-H) bases (Supplementary Fig. 6), and fluorescence values were exported from FlowJo and imported into MATLAB for further analysis.

### Secretion assay

For the BMM experiment in Fig. 5, supernatant for each condition was collected at 3 hps in chilled Eppendorf tubes, clarified by centrifugation (14,000 × *g*, 4 °C, 10 min), pipetted into new chilled tubes, and stored (–80 °C). Clarified supernatants were later thawed on ice, and analytes were measured using the Bio­Plex Pro mouse cytokine 23­-plex Group I assay (Bio-Rad #M60009RDPD), a Bio-Plex 200 instrument, and Milliplex Analyst software. Each biological replicate was assayed in technical duplicate. Protein concentrations were determined based on a standard curve as described by the manufacturer-provided protocol and values for Lot #64209360. This assay reflects analytes that were secreted between the time of plating and time of BFA treatment. For the condition with sTNFR treatment, in which extracellular TNF is bound by sTNFR, low TNF signal is potentially attributable to an inaccessibility for binding by assay detection reagents.

### Data visualization

Flow cytometry distributions are plotted with a logicle x-axis, a standard method that uses linear scaling near the zero mark and log_10_-scaling farther from zero^79^. Confocal trajectories are plotted with linear axes unless stated otherwise. For histogram simulations corresponding to flow cytometry, a small arbitrary constant value was added uniformly to the simulated values, and probability distributions were plotted on a log_10_ *x*-axis; this procedure approximates logicle scaling and facilitates clearer comparisons with data.

### Statistical analysis

In Fig. 2d, e and Fig. 3e, f, *p*-values were determined using one-tailed permutation tests (*n* = 30 cells). Significance in Fig. 2g and Supplementary Fig. 2r was assessed using four-factor ANOVAs and Tukey’s honest significant difference (HSD) tests (Supplementary Note 1). Significance in Fig. 5a, b and Supplementary Fig. 5c was assessed using two-factor ANOVAs and Tukey’s HSD tests (Supplementary Note 2). Effects were considered significant if ANOVA *p* < 0.05 and if comparisons had an adjusted *p* < 0.05.

### Reporting summary

Further information on research design is available in the Nature Research Reporting Summary linked to this article.

## Supplementary information

Supplementary Information

Reporting Summary

Description of Additional Supplementary Files

Supplementary Software 1

## Data Availability

Data are available in supplementary information files and from the corresponding authors upon request. Source data for Fig. 1b–e, Fig. 2a–g, Fig. 4a–c, Fig. 5a, b, Supplementary Fig. 1a–e, Supplementary Fig. 2b–r, Supplementary Fig. 3c, Supplementary Fig. 4a–d, and Supplementary Fig. 5a–d, f are also provided as a Source Data file.

## Source data

Source Data

## Code availability

Model formulation, development, and calibration are described in Supplementary Methods. Code is provided as Supplementary Software 1 and at 10.5281/zenodo.3537942 and https://github.com/leonardlab/MacrophageQL under an open source license^80^.
