# Supplementary material for: Macrophages employ quorum licensing to regulate collective activation
Source: Nat Commun. 2020 Feb 13;11:878. doi: 10.1038/s41467-020-14547-y (PMC7018708; doi:10.1038/s41467-020-14547-y)
Supplement: Supplementary file 1 — Supplementary Information [file 41467_2020_14547_MOESM1_ESM.pdf]

Supplementary Information

Macrophages employ quorum licensing to regulate collective activation

Muldoon et al.

Supplementary Figures

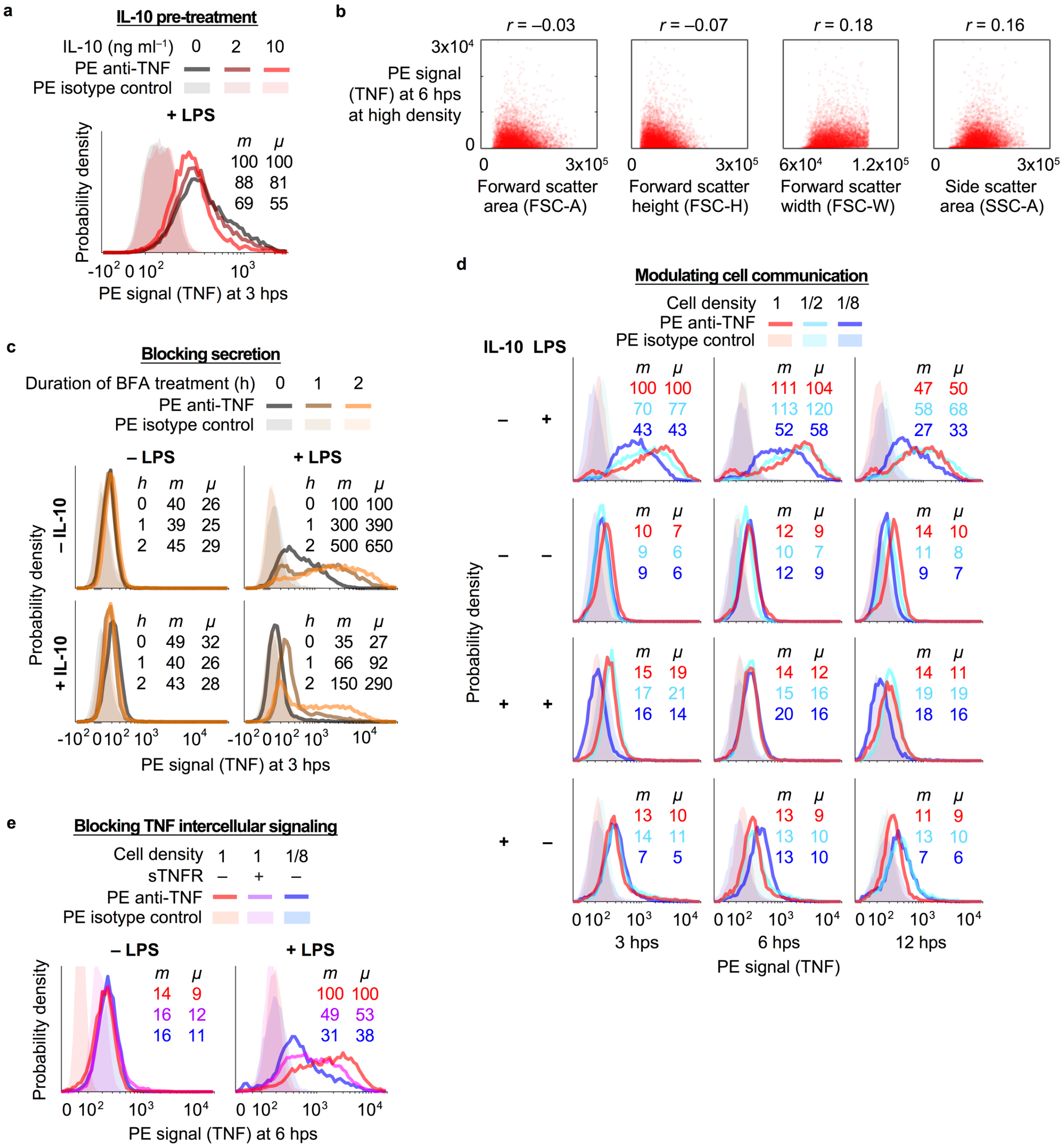

Supplementary Fig. 1 Heterogeneity in macrophage activation. **a** Quantification of the effect of IL-10 pre-treatment in **Fig. 1b**. **b** Low Pearson correlation coefficients (*r*) indicate little effect of flow cytometric proxies for cell size on TNF expression. Axes are in linearly-scaled units. **c** Quantification of the effect of BFA treatment in **Fig. 1c**. **d** Quantification of the effect of cell density in **Fig. 1d**. **e** Quantification of the effect of sTNFR pre-treatment in **Fig. 1e**. For each panel, the median (*m*) and mean (*μ*) were calculated by subtracting the isotype control value and normalizing to the reference condition labeled 100%.

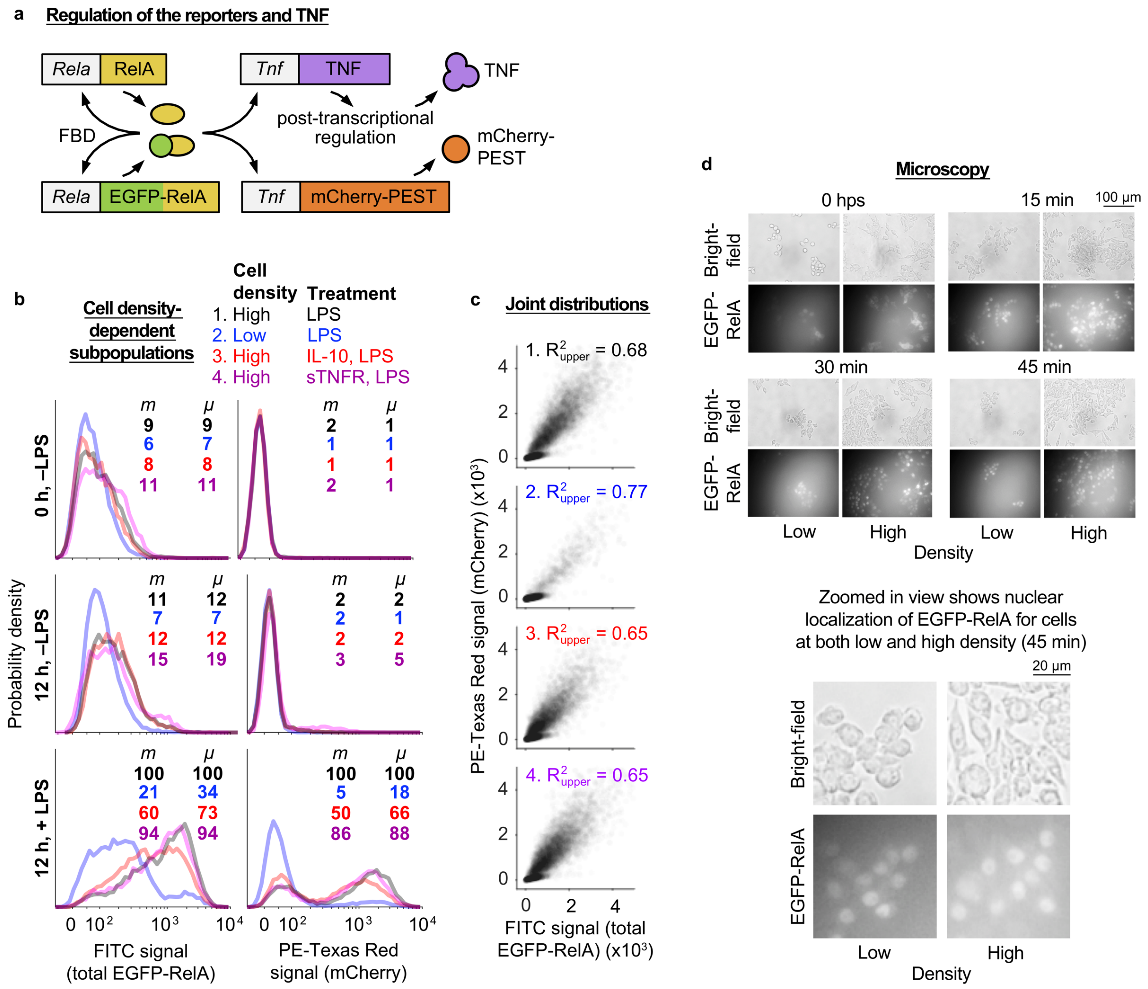

Supplementary Fig. 2 Single-cell and density-associated analysis of activation heterogeneity. a Summary diagram of the regulation of endogenous RelA and TNF and the reporters EGFP-RelA and mCherry. **b** Quantification of reporter signal in **Fig. 2a**. For each reporter, the median (*m*) and mean (*μ*) were calculated by normalizing to the reference condition labeled 100%. X-axes are on a logicle scale. **c** Cells occupy high and low activation states. Data are from the four conditions at 12 hps in **b**. Within the subpopulation that undergoes high activation ("upper"), reporters are correlated highly and similarly across conditions. Axes in **c** are linearly scaled. **d** LPS induces EGFP-RelA nuclear translocation at both high cell density and low (one-eighth) cell density. Representative microscopy images (different cells at each time point) show intracellular localization over time (minutes post-LPS stimulation). EGFP-RelA is initially primarily cytoplasmic, and after LPS treatment, the nuclear signal increases. Translocation occurs at both cell densities, indicating that low density does not prevent TLR4 signaling from activating NF-κB. The scale bar denotes 100 μm in the upper panel and 20 μm in the lower panel.

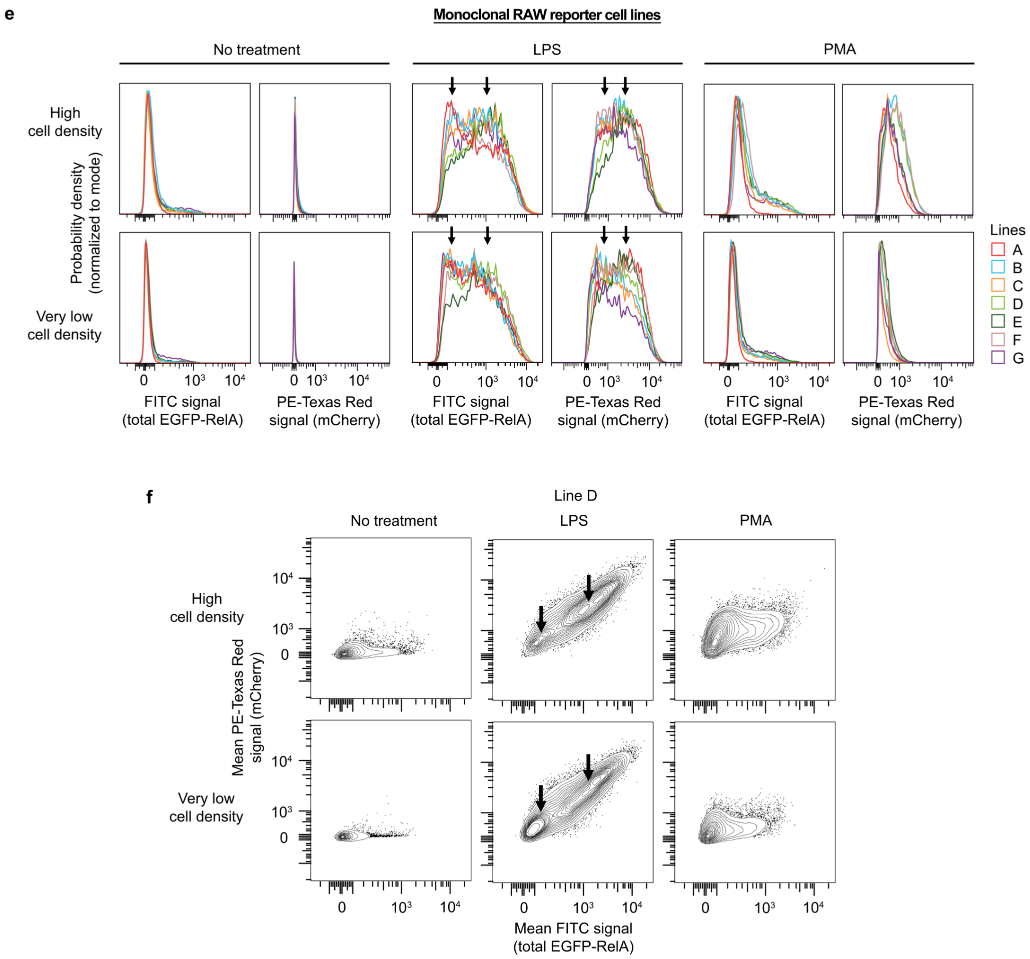

Supplementary Fig. 2. e–i Monoclonal cell lines (A–G, color-coded) were generated from the reporter line. **e–h** Cells from monoclonal lines were plated at high density and very low (1/64) density at –36 hps, treated with LPS (100 ng ml^{-1}), PMA (100 ng ml^{-1}), or neither at 0 hps, and harvested at 12 hps for flow cytometry. **e** Reporter signal shows ligand-induced bimodality. **f** Joint distributions from monoclonal reporter cell line D, with arrows identifying the high and low LPS-induced activation states.

Monoclonal RAW reporter cell lines

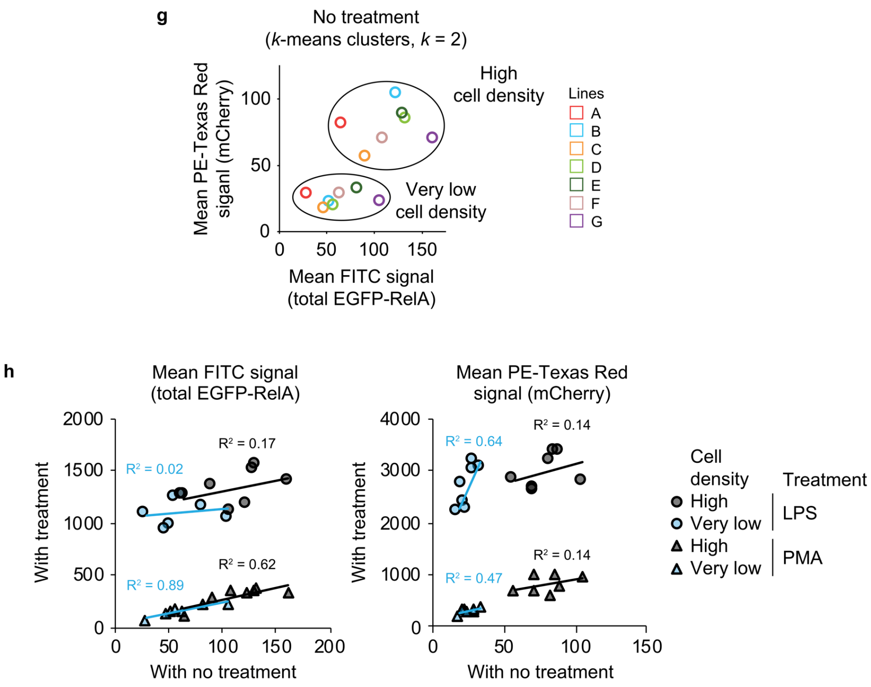

Supplementary Fig. 2. g *k*-means clustering (*k* = 2) on mean total EGFP-RelA signal and mean mCherry signal from untreated lines (A–G, color-coded) accurately clusters resting states for the two cell densities. **h** Comparison of basal and induced expression for each reporter. Data points are the mean signals from each of the seven cell lines. Color-coding is by cell density, and shapes denote comparisons between no treatment and LPS (circles) or between no treatment and PMA (triangles). R^2 values are Pearson correlation coefficients for each regression line (for the four combinations of density and treatment). For some of these combinations, the resting state is highly correlated with the induced state across cell lines.

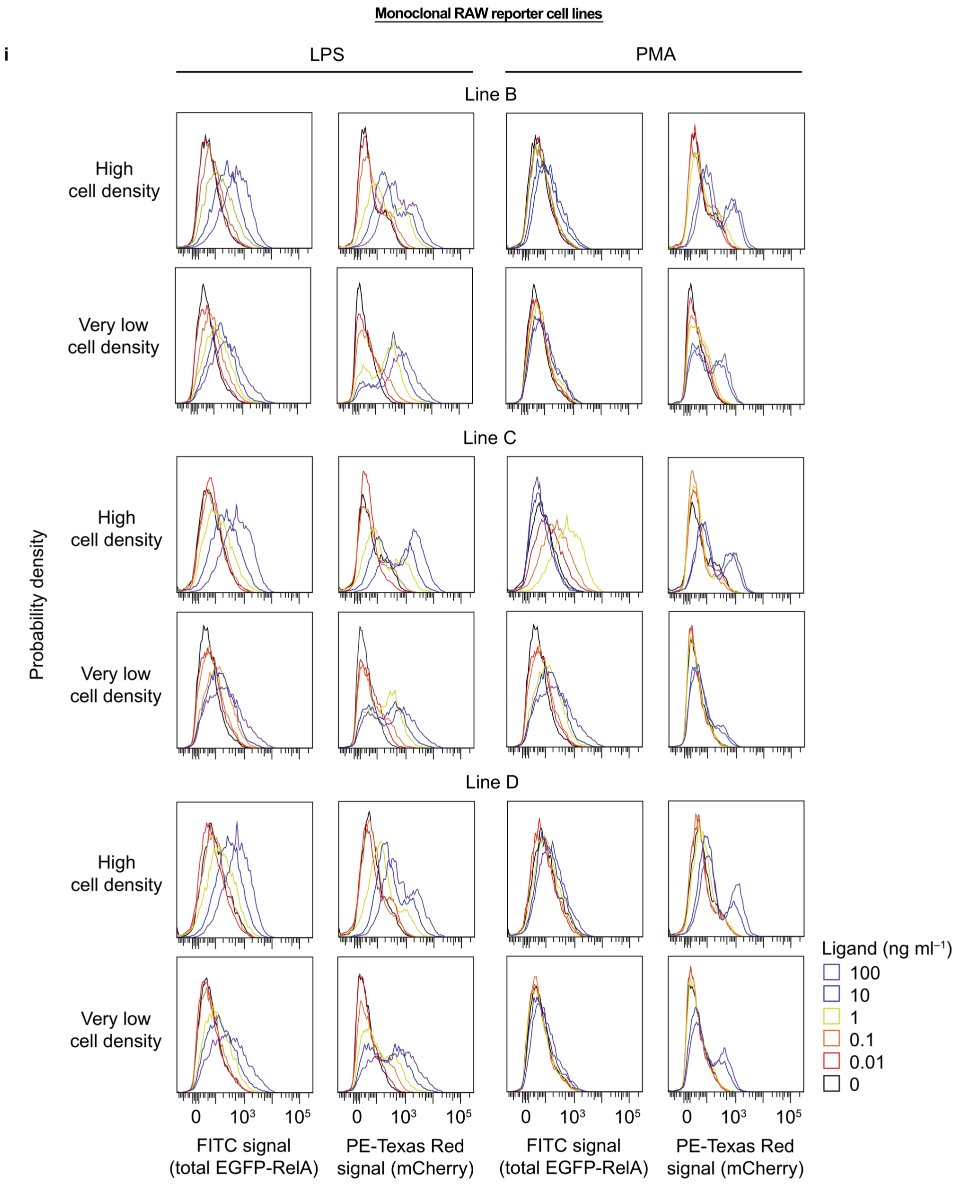

Supplementary Fig. 2. i Cells from three monoclonal reporter cell lines were plated at high and very low density at –36 hps, treated with LPS (doses indicated), PMA (doses indicated), or neither at 0 hps, and harvested at 12 hps for flow cytometry. Trends in reporter signal broadly agree with the observations in **Fig. 4a** for the parental reporter line, from which the monoclonal lines were derived. Ligand-induced bimodality is apparent in each monoclonal cell line and is more pronounced at higher ligand doses.

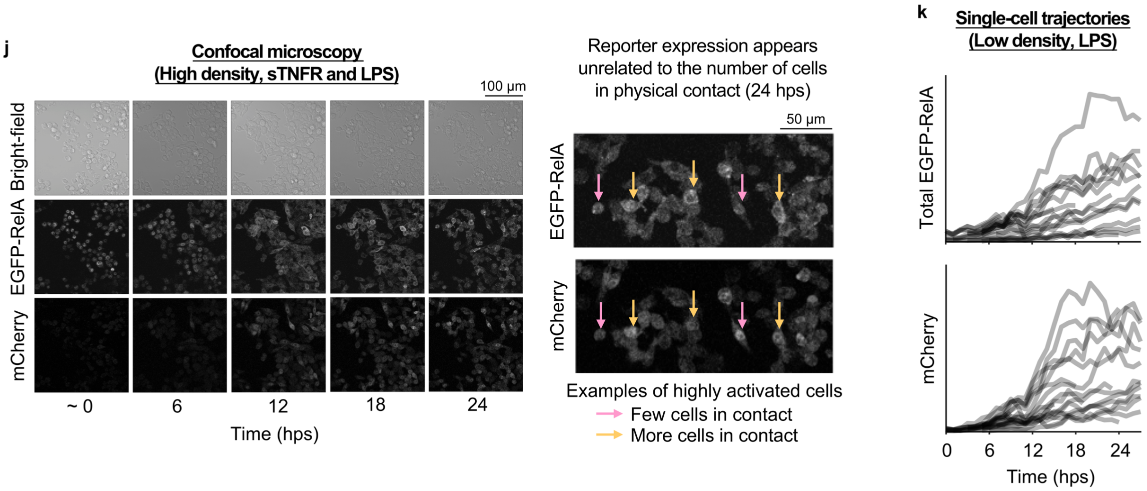

Supplementary Fig. 2. j Cell-to-cell contact does not explain heterogeneous activation. Confocal microscopy images are shown for cells treated with sTNFR and LPS. ~0 indicates a timepoint shortly after LPS treatment. Cells with high fluorescence occur throughout the field of view, and some cells are adjacent to many cells while others are adjacent to few. Patterns were not apparent between a cell's fluorescence and the number of neighboring cells or their fluorescence. Although these snapshots do not capture the continuous history of cell-cell contact, as cells can move over time, the clumped cells show no discernible increase in fluorescence. The scale bar denotes 100 μm in the left panel and 50 μm in the right panel. For purpose of visualization, images in **d** and **j** were brightened without altering the contrast. **k** Confocal microscopy trajectories of total EGFP-RelA and mCherry for 20 cells at low density; some traces do not span the full timecourse due to mitosis or to cells exiting the field of view.

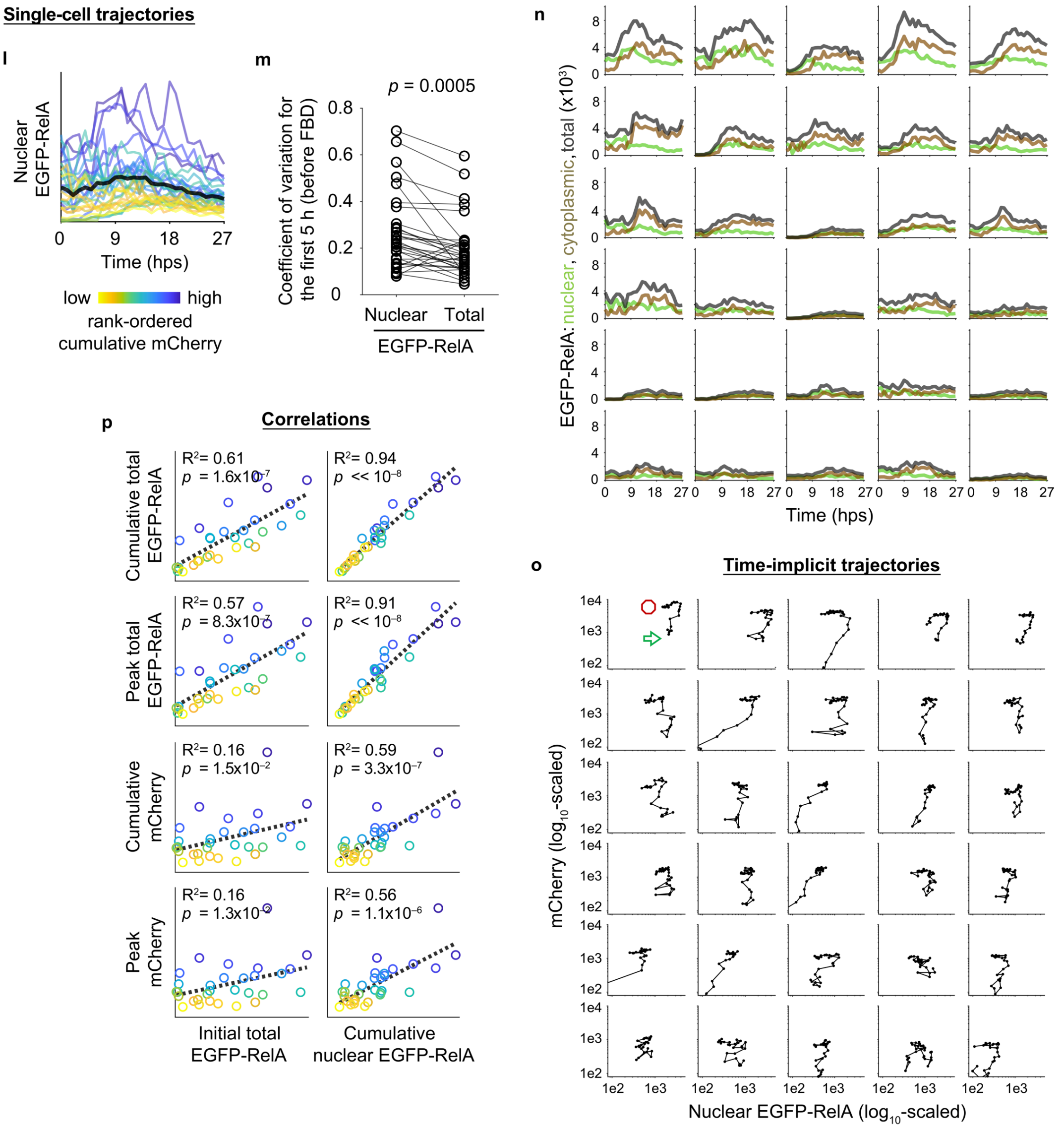

Supplementary Fig. 2. l–p Analysis of reporter trajectories at high density with sTNFR and LPS. **l** Traces are color-coded by rank-ordered cumulative mCherry. **m** The coefficient of variation is greater for nuclear than for total EGFP-RelA, consistent with nucleocytoplasmic translocations. **n** Traces for nuclear (green), cytoplasmic (tan), and total (gray) EGFP-RelA are ordered row-wise from upper-left to lower-right by high-to-low cumulative mCherry. Peak nuclear signal generally precedes peak cytoplasmic signal. Axes are linearly scaled. **o** Time-implicit trajectories of nuclear EGFP-RelA and mCherry vary in activation magnitude but follow a characteristic pattern: starting in the lower-left, moving to the upper-right, and moving to the left. The arrow and octagon indicate the start and finish. Axes are $\log_{10}$-scaled. **p** Initial EGFP-RelA and integrated nuclear EGFP-RelA are predictive for total EGFP-RelA and mCherry. *P*-values are from a one-tailed test for the Pearson correlation. In **l** and **p**, color-coding denotes rank-ordered cumulative mCherry expression.

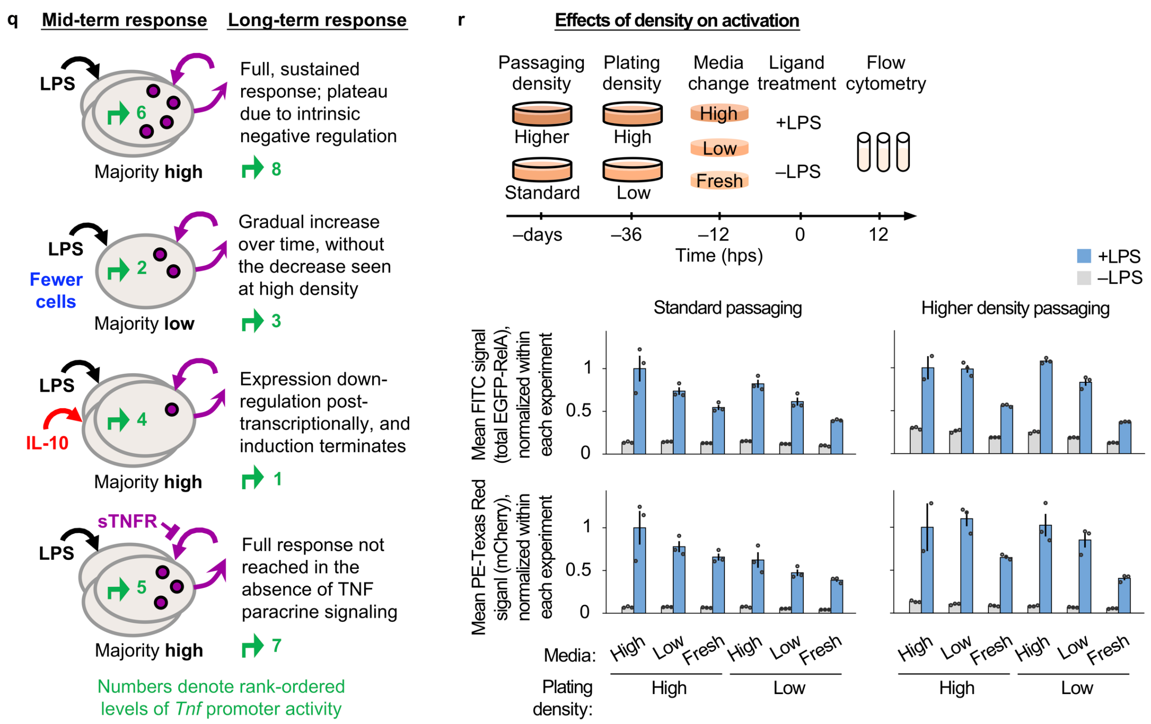

Supplementary Fig. 2. q The diagram summarizes observations on *Tnf* promoter activity (green arrow) and TNF protein expression (purple circles) for mid-term (12 hps) and long-term (24 hps) responses across perturbations. We note that when quantifying reporter expression by microscopy, the range of signals is characteristically narrower than with flow cytometry, and additionally that fluorescence readout is experiment-specific such that the most comparable features are magnitude-independent, e.g., trajectory shape and time to reach peak signal. Therefore, to better enable comparisons, we used mean values in **Fig. 2a** in comparable flow cytometry units to scale the microscopy data in **Fig. 2f** at 12 hps to comparable units from which long-term responses could be estimated. This analysis was used to rank *Tnf* promoter activity based on normalized mCherry signal from low to high (1–8). **r** Modulating intercellular communication through the cell density during passaging, at plating, and for the media change. Data for standard passaging and higher density passaging were collected separately and are in distinct fluorescence units. Data are normalized within each fluorescent reporter readout. Bar graphs represent the mean from three biological replicates and S.E.M. Effects of passaging, cell density, media, and treatment condition were assessed using four-factor ANOVAs and Tukey's HSD tests; outcomes are in **Supplementary Note 1**.

a

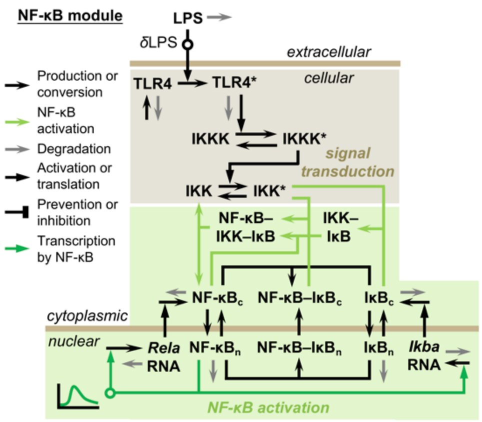

Supplementary Fig. 3 Computational model development. **a** Diagram of the NF-κB module. Arrows denote processes as indicated in the legend. State variables are in bold non-italicized text. **b** Simulations across LPS doses and NF-κB initial values (inactive cytoplasmic fraction), with and without FBD. Base case values are 100 ng ml^{-1} dose and *1x* initial value (0.1 a.u.). The first 16 panels are individual state variables, and the last three panels are the total cytoplasmic, total nuclear, and total-cell amounts of NF-κB (summed from the individual variables).

b

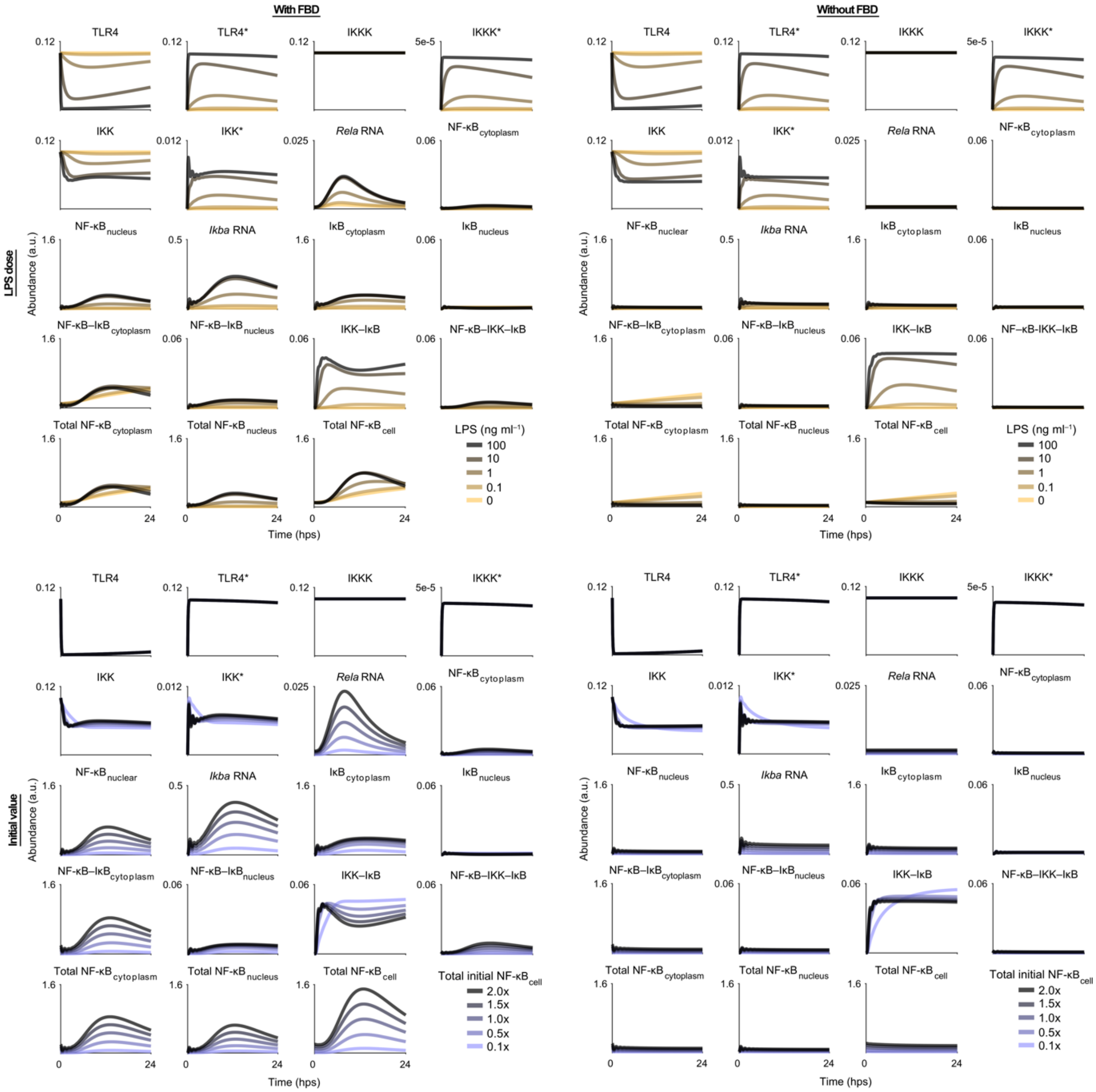

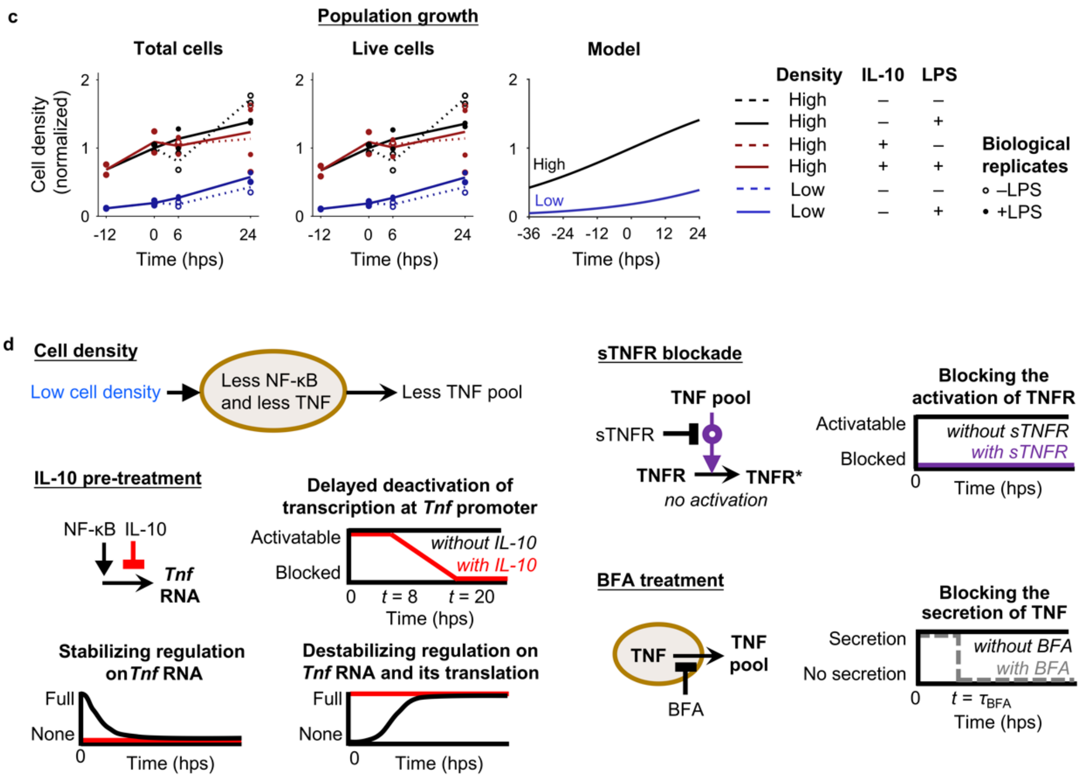

Supplementary Figure 3. c Population growth. RAW cells were plated at high density (3.3×10^5 cells ml^{-1}) or low density (4.1×10^4 cells ml^{-1}) at –36 hps. To quantify density from the same plates across time, representative images were obtained by bright-field microscopy at several time points, and these images underwent automated analysis in ImageJ. Plots represent the mean and one standard deviation, as determined from five images from each of two replicate plates. Y-axis units are normalized such that high density at 0 h is defined as 1 a.u. *Left:* growth is similar with and without IL-10 pre-treatment or LPS treatment. *Middle:* cell viability as estimated from Trypan blue staining was approximately 100% across conditions. *Right:* fitted model for cell growth as a function of time and the density at plating. **d** The diagrams summarize how each treatment's or perturbation's mode of action is represented in the model. Ovals represent cells. Low cell density leads to a decrease in LPS-induced TNF secretion. IL-10 pre-treatment inhibits sustained LPS-induced activation at the *Tnf* promoter and decreases *Tnf* mRNA half-life via removal of stabilizing regulation and promotion of destabilizing regulation. sTNFR pre-treatment blocks TNF intercellular signaling. BFA treatment prevents secretion, leading to intracellular TNF accumulation.

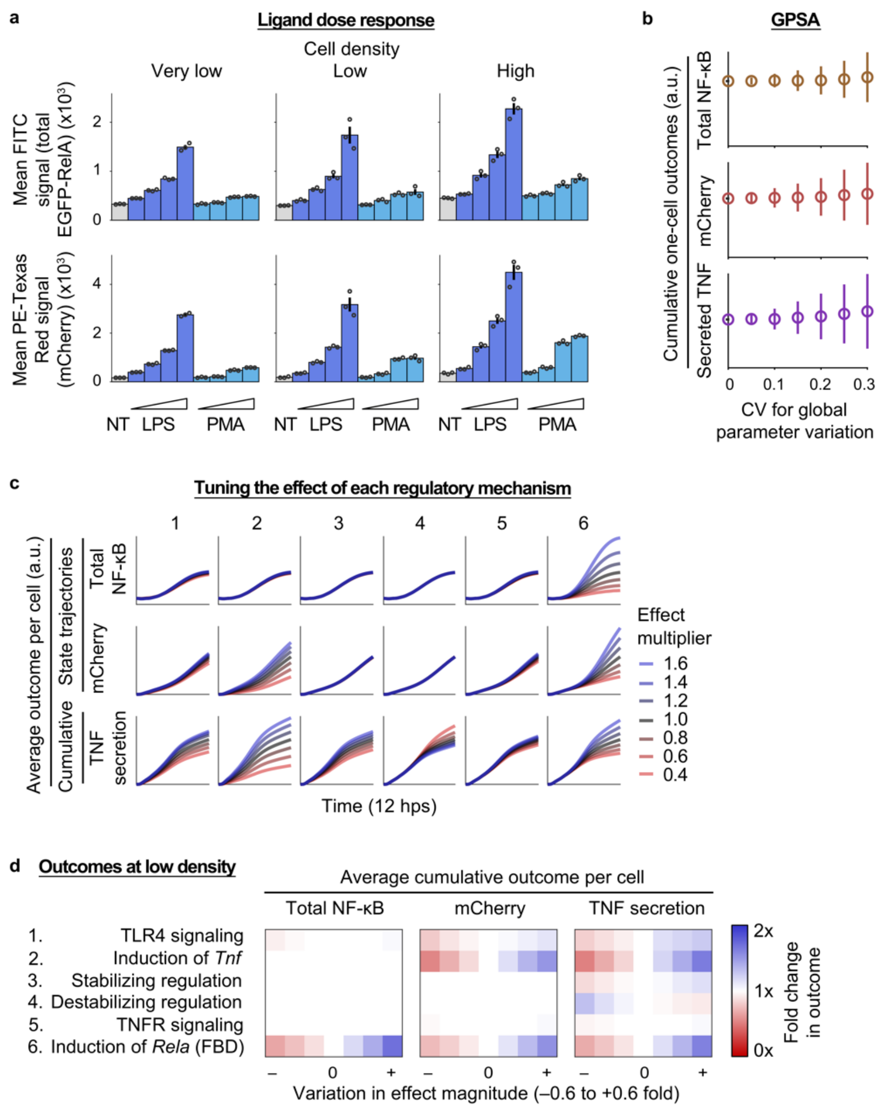

Supplementary Fig. 4 Model analysis. a Flow cytometry data from **Fig. 4a** for reporter signal at 12 h post-LPS or post-PMA (0.1, 1, 10, and 100 ng ml^{-1}). Bar graphs represent the mean of three biological replicates and S.E.M. **b** Global parameter sensitivity analysis (GPSA). Values for the twelve estimated free parameters were varied simultaneously by randomly sampling from Gaussian distributions centered on the true-estimated values with increasing coefficients of variation (CV; x-axis). Homogeneous simulations were run at high density. Outcomes are summarized by three metrics that involve integrating values from 0–12 hps: total NF-κB, mCherry, and secreted TNF (flux). Y-axes are in linearly scaled units specific to each plot and are normalized to base case values (at CV = 0). Line indicate +/– one standard deviation from the mean of 1,000 random samples per CV. The outcomes are robust to global parameter variation up to ~0.1–0.2 CV. **c** Outcomes from varying the effect magnitude (color-coded) for each mechanism in **Fig. 4b**. Population-mean reporter trajectories and cumulative secreted TNF are shown for a heterogeneous population at high density (0–12 hps). Cumulative secreted TNF is the total secretion by a given time point, determined by integrating the flux of secretion over time. Y-axes are in linearly scaled units and are specific to each row of plots. **d** Analogous analysis to **Fig. 4b** for low density population. Trends resemble those at high density.

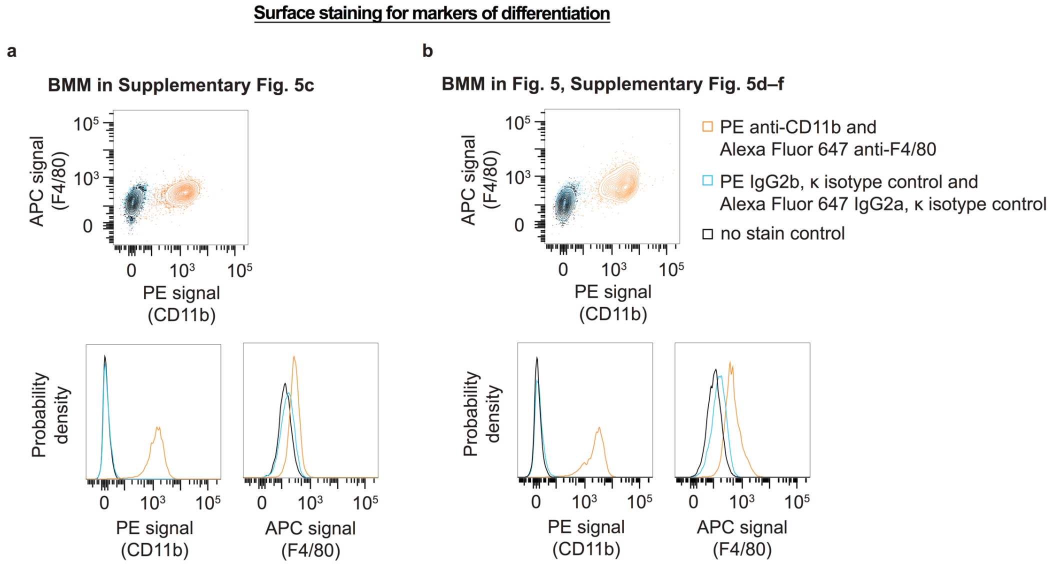

Supplementary Fig. 5 Primary cell experiments. a–b Surface staining for markers of differentiation. At seven days after each of the two bone marrow harvests, differentiation to macrophages was assessed by surface staining for CD11b and F4/80. A signal that is distinguishable from the isotype control was observed for both harvests.

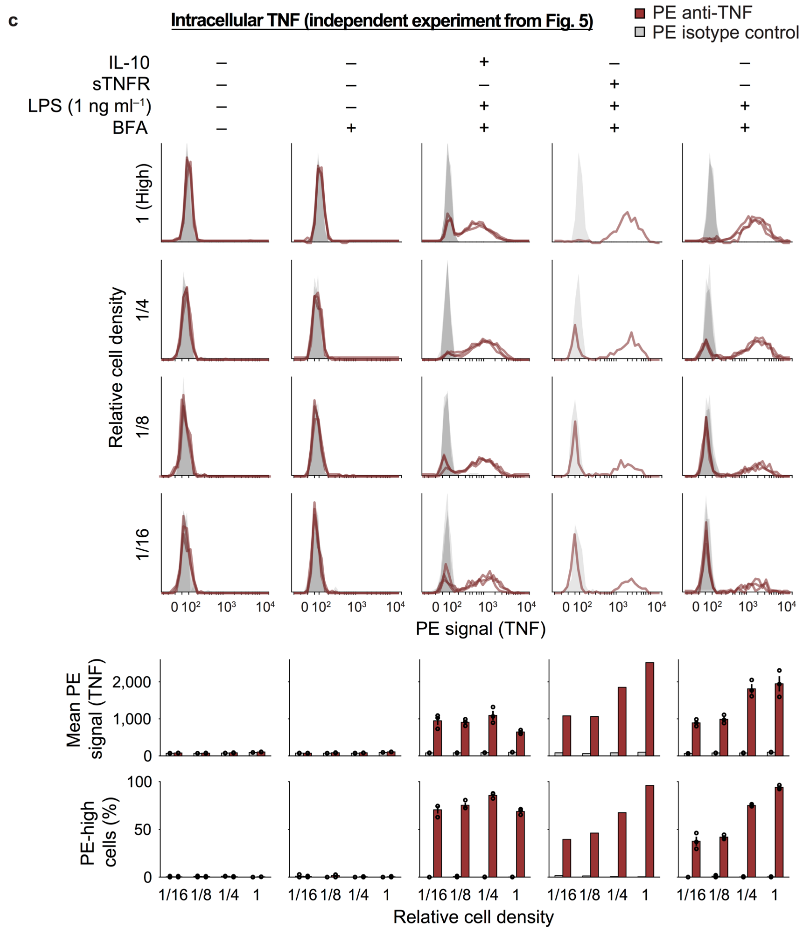

Supplementary Fig. 5. c An experiment varying the plating density and treatment conditions shows cell density-dependent bimodality in LPS-inducible TNF expression. This panel represents an experiment that is independent from that in **Fig. 5a**. Treatment conditions (columns) were sampled in biological triplicate, except for the fourth condition (with sTNFR), which was sampled using one biological replicate. Timing and doses of ligands are described in **Methods**. Bar graphs represent the mean of the biological replicates and S.E.M. Outcomes from two-way ANOVAs and Tukey's HSD tests are in **Supplementary Note 2**.

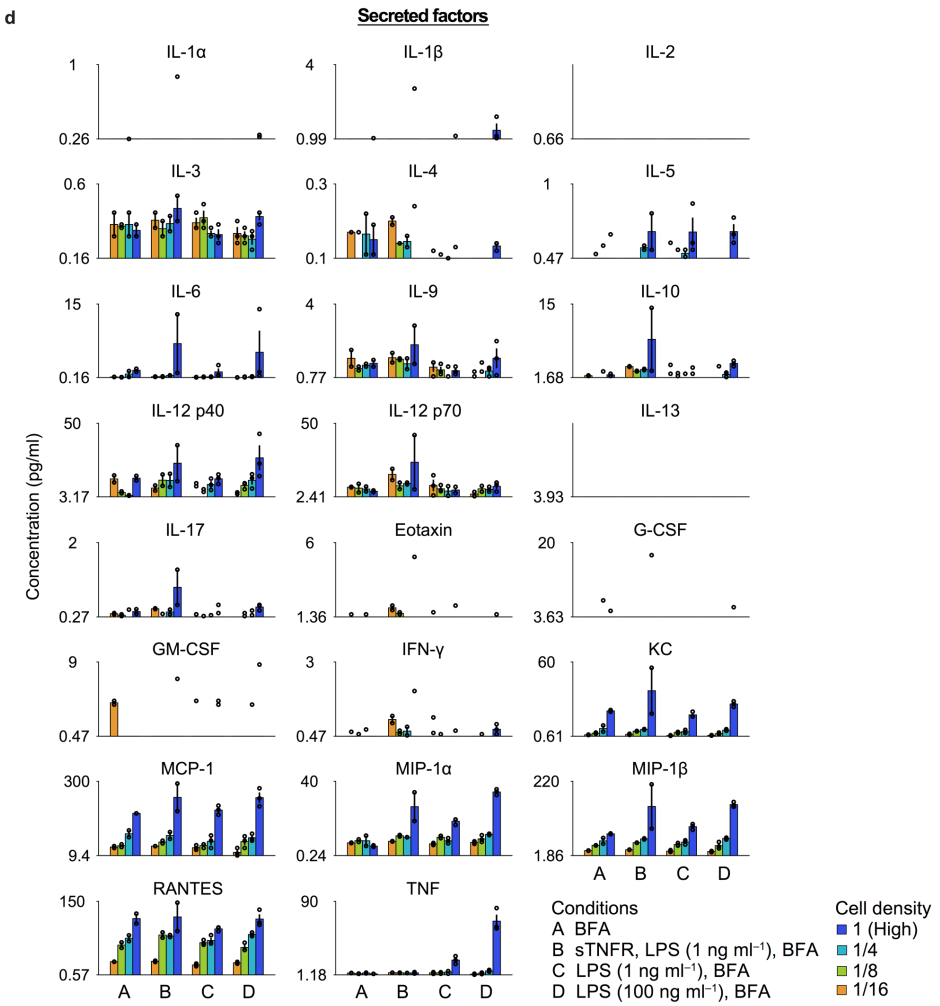

Supplementary Fig. 5. d Secreted factors in the supernatant were evaluated by multiplexed assay (23 analytes). These cell culture supernatants correspond to the experiment in **Fig. 5**. Bars graphs represent the mean of the biological replicates and S.E.M. The minimum value on each y-axis is the observed lower limit of detection of the assay. For each analyte-density-treatment combination, if at least one biological replicate was below the limit of detection, then the mean for the set of replicates was considered to be below the limit. Outcomes from two-way ANOVAs and Tukey's HSD tests are in **Supplementary Note 2**.

e

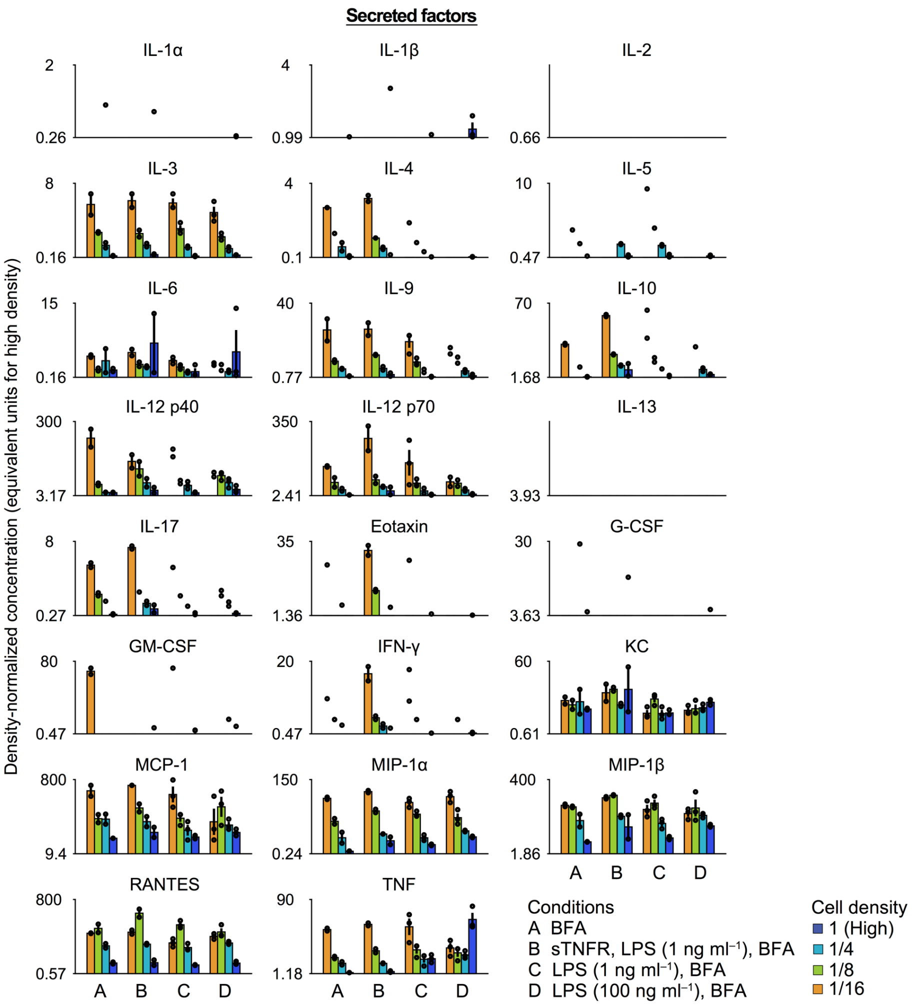

f

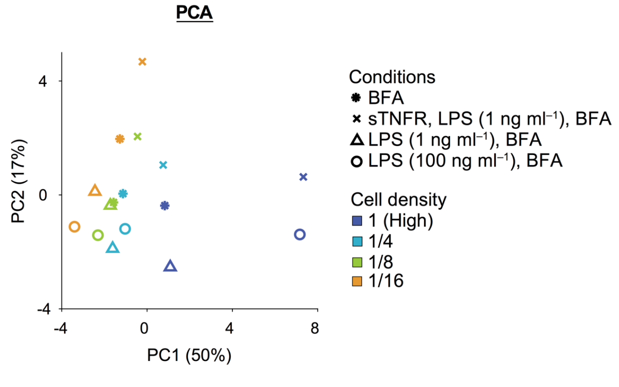

Supplementary Fig. 5. e Data for the multiplexed assay in panel **d** are shown using scaled values for the mean, S.E.M., and biological replicates all in arbitrary units—dividing each value by the relative cell density at the time of plating (1/16, 1/8, 1/4, or 1). **f** Principle component analysis on data from panel **d**, for 23 analyte variables across 16 observations (four cell densities, four treatment conditions). The first two principle components are shown, and the percent of variation captured by each is in parentheses. The first principle component captures much of the variation associated with cell density.

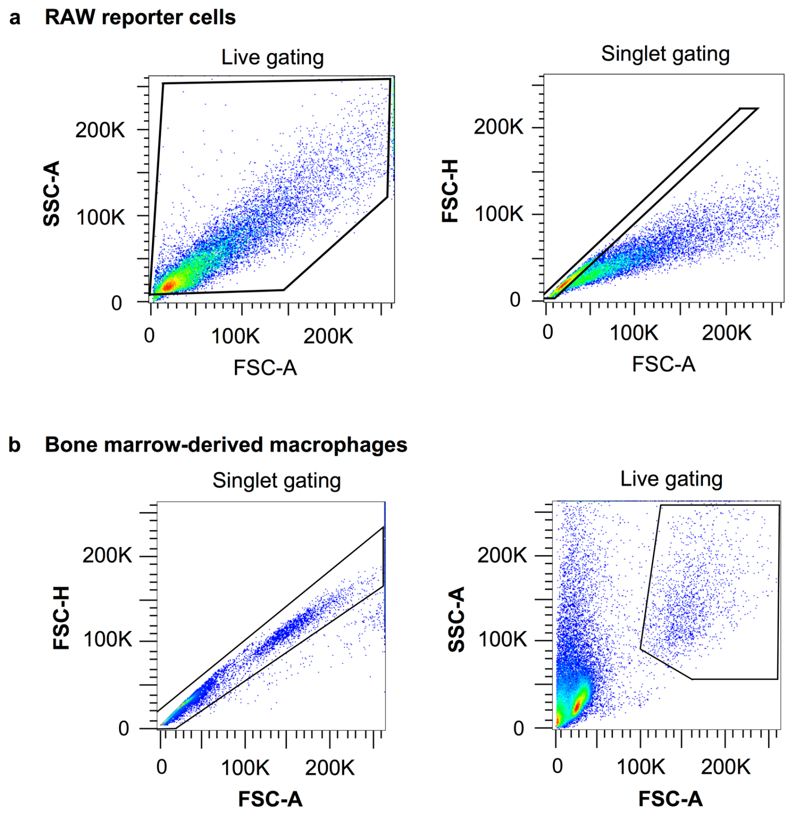

Supplementary Fig. 6. Gating strategies. Cells were gated on live (FSC-A vs. SSC-A) and single-cell (FSC-A vs. FSC-H) bases. **a** RAW cells from Fig. 1, and **b** primary cells from Fig. 5. The gating strategy for RAW cells was also used for RAW reporter cells.

Supplementary Tables

Supplementary Table 1. State variables

# [a]	Names	Descriptions	Initial values (a.u.)
$\dot{x}_1$	**TLR4**	Inactive TLR4	0.1
$\dot{x}_2$	**TLR4***	Active TLR4	0
$\dot{x}_3$	**TNFR**	Inactive TNFR	0.1
$\dot{x}_4$	**TNFR***	Active TNFR	0
$\dot{x}_5$	**IKKK**	Inactive	0.1
$\dot{x}_6$	**IKKK***	Active	0
$\dot{x}_7$	**IKK**	Inactive	0.1
$\dot{x}_8$	**IKK***	Active	0
$\dot{x}_9$	**NFkBm**	*Rela* mRNA	varies by cell [a]
$\dot{x}_{10}$	**NFkBc**	NF-κB cytoplasmic	0
$\dot{x}_{11}$	**NFkBn**	NF-κB nuclear	0
$\dot{x}_{12}$	**IkBm**	*Ikba* mRNA	0
$\dot{x}_{13}$	**IkBc**	IκB cytoplasmic	0
$\dot{x}_{14}$	**IkBn**	IκB nuclear	0
$\dot{x}_{15}$	**NFkB_IkBc**	NF-κB–IκB cytoplasmic	varies by cell [b]
$\dot{x}_{16}$	**NFkB_IkBn**	NF-κB–IκB nuclear	0
$\dot{x}_{17}$	**IKK_IkB**	IKK–IκB	0
$\dot{x}_{18}$	**NFkB_IKK_IkB**	NF-κB–IKK–IκB	0
$\dot{x}_{19}$	**Stabilizing_regulation**	Stabilizing regulation	1 without IL-10; 0 with IL-10
$\dot{x}_{20}$	**Destabilizing_regulation**	Destabilizing regulation	0 without IL-10; max. with IL-10
$\dot{x}_{21}$	**mCherrym**	*mCherry* mRNA	0
$\dot{x}_{22}$	**mCherry**	mCherry protein	0
$\dot{x}_{23}$	**mCherryf**	mCherry protein folded	0
$\dot{x}_{24}$	**Tnfm**	*Tnf* mRNA	0
$\dot{x}_{25}$	**TNF**	TNF protein	0
$\dot{x}_{EC1}$ [c]	**TNFpool**	Extracellular TNF protein	0
$\dot{x}_{EC2}$ [c]	**LPS**	LPS stimulus	1 (corresponds to 100 ng/ml)

[a] Variables #1 through 25 are intracellular, and the last two variables are extracellular.
ODEs for one cell are in **Supplementary Table 4**. A system of 30 cells has 25x30+2 = 752 ODEs.

[b] Estimated steady-state initial value for *Rela* mRNA: NFkBm = 0.007 * NFkB_IkBc

[c] Initial values of the inactive cytoplasmic complex, based on experimental quantification of of initial EGFP-RelA from confocal microscopy, and normalized so that the mean is 0.1.

NFkB_IkBc = [0.0022, 0.0046, 0.0065, 0.0068, 0.0085, 0.0146, 0.0355, 0.0437, 0.0460, 0.0465, 0.0619, 0.0667, 0.0668, 0.0676, 0.0826, 0.0940, 0.1023, 0.1151, 0.1180, 0.1320, 0.1417, 0.1533, 0.1543, 0.1594, 0.1689, 0.1775, 0.2052, 0.2216, 0.2418, 0.2540]

Imputed values for low density.

NFkB_IkBc = [0.0016, 0.0034, 0.0049, 0.0051, 0.0064, 0.0109, 0.0266, 0.0328, 0.0180, 0.0299, 0.0130, 0.0305, 0.0326, 0.0058, 0.0069, 0.0349, 0.0500, 0.0507, 0.0620, 0.0705, 0.0767, 0.0885, 0.0990, 0.1063, 0.1157, 0.1267, 0.1331, 0.1539, 0.1662, 0.1813]

[d] The two extracellular (EC) variables are appended to the end of the system of ODEs for one or more cells.

Supplementary Table 2. Parameters

Names	Descriptions	Values	Units [a]	Sources
$k_{synthesis\_TLR4}$	synthesis of TLR4	0.0185	μM h^{-1}	Calculated based on degradation rate
$k_{activate\_TLR4}$	activation of TLR4	10	$μM^{-1}$ h^{-1}	Estimated, to produce oscillatory translocations at 100 ng/ml LPS and lesser effects at lower doses
k_{deg_TLR4}	degradation of TLR4	0.185	h^{-1}	Estimated from a reduced model of Cheng et al. 2015[1]
$k_{synthesis\_TNFR}$	synthesis of TNFR	0.0102	μM h^{-1}	Calculated based on degradation rate
$k_{activate\_TNFR}$	activation of TNFR	[b] 0.614 / nc	$μM^{-1}$ h^{-1}	Fitted
k_{deg_TNFR}	degradation of TNFR	0.102	h^{-1}	From Werner et al.[16] and used by Caldwell et al.[17]
k_{TLR4_IKKK}	activation of IKKK by TLR4	0.588	$μM^{-1}$ h^{-1}	Estimated in order to produce oscillatory translocations
k_{TNFR_IKKK}	activation of IKKK by TNFR	0.588	$μM^{-1}$ h^{-1}	Assumed equal to kTLR4_IKKK
$k_{inactivate\_IKKK}$	inactivation of IKKK	150	h^{-1}	Cheng et al. 2015[1]
$k_{activate\_IKK}$	activation of IKK	60000	$μM^{-1}$ h^{-1}	Cheng et al. 2015[1]
$k_{inactivate\_IKK}$	inactivation of IKK	19.4	h^{-1}	[c] Fitted in the NF-κB module
$k_{assoc\_IKK\_IkBc}$	association of IKK and IκBc	81	$μM^{-1}$ h^{-1}	Cheng et al. 2015[1]
$k_{assoc\_IKK\_NFkBIkBc}$	association of IKK and NF-κB–IκBc	666	$μM^{-1}$ h^{-1}	Cheng et al. 2015[1]
$k_{deg\_NFkBIkBIKKc}$	degradation of NF-κB–IκB–IKKc	432	h^{-1}	Cheng et al. 2015[1]
k_{tx}	max. transcription	1	μM h^{-1}	Assumed equal for all genes
$w_{basaltx\_NFkB}$	basal transcription of NF-κB mRNA	[d] mean=10^{-4}	μM	Estimated to maintain NF-κB expression in absence of FBD
w_{NFkBtx_NFkB}	transcription of NF-κB mRNA by NF-κB	0.219	N/A	[c] Fitted in the NF-κB module
w_{IL10tx_FBD}	repression of transcription of NF-κB mRNA by NF-κB, by IL-10	Fitted: 7.27	μM	[c] Fitted in the NF-κB module
k_{deg_NFkBm}	degradation of NF-κB mRNA	0.14	h^{-1}	Assumed 5 h half-life
k_{tl}	translation	15	h^{-1}	Cheng et al. 2015[1]
k_{import_NFkBc}	import of NF-κB from the cytoplasm to the nucleus	324	h^{-1}	Cheng et al. 2015[1]
k_{export_NFkBn}	export of NF-κB from the nucleus to the cytoplasmic	1.5	h^{-1}	Estimated to produce oscillatory translocations
$k_{assoc\_NFkB\_IkB}$	association of NF-κB and IκB	1800	μM h^{-1}	Cheng et al. 2015[1]
$k_{assoc\_IkBIKKc\_NFkBc}$	association of IκB–IKKc and NF-κBc	1800	μM h^{-1}	Cheng et al. 2015[1]

k_{deg_NFkB}	degradation of NF-κB	0.36	h^{-1}	Matched to the estimate for IκB in the resting state (where IKK is inactive); Sung et al.[4]
w_{NFkBtx_IkB}	transcription of IκB mRNA by NF-κB	6.53	N/A	[c] Fitted in the NF-κB module
k_{deg_IkBm}	degradation of IκB mRNA	2.1	h^{-1}	Cheng et al. 2015[1]
k_{import_IkBc}	import of IκBc from the cytoplasm to the nucleus	1.08	h^{-1}	Cheng et al. 2015[1]
k_{export_IkBn}	export of IκBn from the nucleus to the cytoplasmic	0.72	h^{-1}	Cheng et al. 2015[1]
k_{deg_IkB}	degradation of IκB	4.2	h^{-1}	Cheng et al. 2015[1]
$k_{export\_NFkBIkBn}$	export of NF-κB–IκBn	49.7	h^{-1}	Kalita et al. 2011[18]
$tau_{stabilize}$	timing of stabilizing regulation ending	0.234	h^{-1}	Fitted
tau_{IKKK}	contribution of IKKK activity to stabilizing regulation	$3.96 * 10^{5}$	μM^{-1}	Fitted
$c_{stabilize}$	threshold IKKK activity for stabilizing regulation	$3.92 * 10^{-5}$	μM	Estimated; involved in stabilizing regulation, and is based on the values of the IKKK* state variable
$tau_{destabilize}$	timing of destabilizing regulation starting	7.3	h	Fitted
w_{NFkBtx_Tnf}	transcription of Tnf mRNA & mCherry mRNA by NF-κB	1.99	N/A	Fitted
$k_{deg\_mCherrym}$	degradation of mCherry mRNA	2.1	h^{-1}	Assumed equal to half-life of other short-lived mRNAs
$k_{deg\_mCherry}$	degradation of mCherry	0.5	h^{-1}	Assumed half-life near 1 h; estimated based on avg. time to peak expression
k_{mature}	maturation (folding) of mCherry	0.7	h^{-1}	Assumed half-life for maturation of 1 h
k_{deg_Tnfm}	degradation of Tnf mRNA	2.1	h^{-1}	Assumed equal to half-life of other short-lived mRNAs
$w_{stabilize}$	effect of stabilizing regulation	1.7	μM^{-1}	Fitted
$w_{maxdestabilize}$	maximum stabilizing regulation	3	N/A	Estimated constant involved in destabilizing regulation
$w_{destabilize}$	effect of destabilizing regulation	0.747	μM^{-1}	Fitted
$k_{secretion}$	TNF secretion	4.17	h^{-1}	Fitted
k_{deg_TNF}	degradation of TNF (intracellular)	0.7	h^{-1}	[e] See note
r_1	max. relative population density	1.99	RCD	[f] Separately fitted
r_2	population growth rate	0.0365	h^{-1}	[f] Separately fitted
$k_{deg\_TNFpool}$	degradation of TNF pool	0.27	h^{-1}	Maiti et al. 2015[19]
k_{deg_LPS}	degradation of LPS (in media)	0.058	h^{-1}	Assumed half-life of 12 h

[a] Units are: concentration (μM), time in hours (h), relative cell density (RCD), number of cells (nc), or no units (N/A).

[b] The parameter for TNFR activation was scaled by dividing by the number of cells. For nc=30, the value is 0.0206 μM^{-1} h^{-1}.

[c] Four parameters were first fit in the NF-κB module; the other eight were fit in the full model.

[d] Varies by cell; equal to 0.001 times the initial concentration of total cellular NF-κB.

[e] We found that given the data available, it would be challenging to estimate values for parameters representing both possible fates of the TNF protein (degradation and secretion). Since degradation is first order and has no downstream consequence in the model, whereas secretion does have a consequence in the activation of TNFR signaling, we chose to prioritize the estimate for secretion. To constrain the search for the free parameter for secretion, we set the value for intracellular degradation to that also used for extracellular degradation (which for simulated outcomes should affect the histograms in **Fig. 3–4** in a linearly proportional manner, and not affect the conclusions).

[f] Separately fitted by constrained nonlinear least squares optimization, prior to fitting the ODE model.

Supplementary Table 3. Stimulus-specific and perturbation-specific parameters

Names	Descriptions	Values	Units
d_{LPS}	LPS treatment at 0 h	1 with; 0 without	N/A
d_{sTNFR}	sTNFR pre-treatment at –1 h	1 with; 0 without	N/A
d_{IL10}	IL-10 pre-treatment at –12 h	1 with; 0 without	N/A
tau_{IL10_1}	time when transcription of Tnf begins to decrease due to IL-10	6	h
tau_{IL10_2}	time when transcription of Tnf effectively ceases due to IL-10	18	h
tau_{BFA}	time of BFA treatment	varies with; infinity without	h
$tau_{density}$	parameter for initial cell density at time of plating (at –36 h)	–0.247 high density; 62.6 low density	h

Supplementary Table 4. Ordinary differential equations

#	ODEs	Reactions
$\dot{x}_1$	$k_{synthesis\_TLR4}$	synthesis: 0 → TLR4
	$- d_{LPS} * k_{activate\_TLR4} * [TLR4] * [LPS]$	activation: TLR4 → TLR4*
	$- k_{deg\_TLR4} * [TLR4]$	degradation: TLR4 → 0
$\dot{x}_2$	$d_{LPS} * k_{activate\_TLR4} * [TLR4] * [LPS]$	activation: TLR4 → TLR4*
	$- k_{deg\_TLR4} * [TLR4^*]$	degradation: TLR4* → 0
$\dot{x}_3$	$k_{synthesis\_TNFR}$	synthesis: 0 → TNFR
	$- k_{activate\_TNFR} * [TNFR] * [TNFpool] * (1 - d_{sTNFR})$	activation: TNFR → TNFR*
	$- k_{deg\_TNFR} * [TNFR]$	degradation: TNFR → 0
$\dot{x}_4$	$k_{activate\_TNFR} * [TNFR] * [TNFpool] * (1 - d_{sTNFR})$	activation: TNFR → TNFR*
	$- k_{deg\_TNFR} * [TNFR^*]$	degradation: TNFR* → 0
$\dot{x}_5$	$- k_{TLR4\_IKKK} * [TLR4^*] * [IKKK]$	activation: IKKK → IKKK*, via TLR4*
	$- k_{TNFR\_IKKK} * [TNFR^*] * [IKKK]$	activation: IKKK → IKKK*, via TNFR*
	$+ k_{inactivate\_IKKK} * [IKKK^*]$	inactivation: IKKK* → IKKK
$\dot{x}_6$	$k_{TLR4\_IKKK} * [TLR4^*] * [IKKK]$	activation: IKKK → IKKK*, via TLR4*
	$+ k_{TNFR\_IKKK} * [TNFR^*] * [IKKK]$	activation: IKKK → IKKK*, via TNFR*
	$- k_{inactivate\_IKKK} * [IKKK^*]$	inactivation: IKKK* → IKKK
$\dot{x}_7$	$- k_{activate\_IKK} * [IKKK^*] * [IKK]$	activation: IKK → IKK*
	$+ k_{inactivate\_IKK} * [IKK^*]$	inactivation: IKK* → IKK
$\dot{x}_8$	$k_{activate\_IKK} * [IKKK^*] * [IKK]$	activation: IKK → IKK*
	$- k_{inactivate\_IKK} * [IKK^*]$	inactivation: IKK* → IKK
	$- k_{assoc\_IKK\_IkBc} * [IKK^*] * [IkBc]$	association: IKK* + IkBc → IKK-IkBc
	$- k_{assoc\_IKK\_NFkBIkBc} * [IKK^*] * [NFkB\_IkBc]$	association: IKK* + NFkB-IkBc → NFkB-IkB-IKKc
	$+ k_{deg\_NFkBIkBIKKc} * [NFkB\_IKK\_IkB]$	degradation: NFkB-IkB-IKKc → IKK + NFkBc
$\dot{x}_9$	$k_{tx} * (w_{basaltx\_NFkB} + d_{LPS} * t\text{^}3 * \exp(-t) / 6 * w_{NFkBtx\_NFkB} * [NFkBn]) / (1 + d_{LPS} * t\text{^}3 * \exp(-t) / 6 * w_{NFkBtx\_NFkB} * ([NFkBn] + w_{IL10tx\_FBD} * d_{IL10}))$	transcription: 0 → NFkBm
	$- k_{deg\_NFkBm} * [NFkBm]$	degradation: NFkBm → 0
$\dot{x}_{10}$	$k_{tl} * [NFkBm]$	translation: 0 → NFkBc
	$- k_{import\_NFkBc} * [NFkBc]$	import: NFkBc → NFkBn
	$+ k_{export\_NFkBn} * [NFkBn]$	export: NFkBn → NFkBc
	$- k_{assoc\_NFkB\_IkB} * [NFkBc] * [IkBc]$	association: NFkBc + IkBc → NFkB-IkBc
	$- k_{assoc\_IkBIKKc\_NFkBc} * [NFkBc] * [IKK\_IkB]$	association: IkB-IKKc + NFkBc → NFkB-IkB-IKKc
	$+ k_{deg\_NFkBIkBIKKc} * [NFkB\_IKK\_IkB]$	degradation: NFkB-IkB-IKKc → IKK* + NFkBc
	$- k_{deg\_NFkB} * [NFkBc]$	degradation: NFkBc → 0
$\dot{x}_{11}$	$k_{import\_NFkBc} * [NFkBc]$	import: NFkBc → NFkBn
	$- k_{export\_NFkBn} * [NFkBn]$	export: NFkBn → NFkBc
	$- k_{assoc\_NFKB\_IkB} * [NFkBn] * [IkBn]$	association: NFkBn + IkBn → NFkB-IkBn
	$- k_{deg\_NFkB} * [NFkBn]$	degradation: NFkBc → 0
$\dot{x}_{12}$	$k_{tx} * w_{NFkBtx\_IkB} * ([NFkBn]) / (1 + w_{NFkBtx\_IkB} * [NFkBn])$	transcription: 0 -> IkBm
	$- k_{deg\_IkBm} * [IkBm]$	degradation: IkBm -> 0
$\dot{x}_{13}$	$k_{tl} * [IkBm]$	translation: IkBm → IkBc
	$- k_{import\_IkBc} * [IkBc]$	import: IkBc → IkBn
	$+ k_{export\_IkBn} * [IkBn]$	export: IkBn → IkBc
	$- k_{deg\_IkB} * [IkBc]$	degradation: IkBc → 0
	$- k_{assoc\_NFkB\_IkB} * [NFkBc] * [IkBc]$	association: NFkBc + IkBc → NFkB-IkBc
	$- k_{assoc\_IKK\_IkBc} * [IKK^*] * [IkBc]$	association: IKK* + IkBc → IKK-IkBc

$\dot{x}_{14}$	k_{import_IkBc} * [IkBc]<br>- k_{export_IkBn} * [IkBn]<br>- k_{deg_IkB} * [IkBn]<br>- $k_{assoc\_NFKB\_IkB}$ * [NFkBn] * [IkBn]	import: IkBc → IkBn<br>export: IkBn → IkBc<br>degradation: IkBn → 0<br>association: NFkBn + IkBn → NFkB-IkBn
$\dot{x}_{15}$	$k_{export\_NFkBIkBn}$ * [NFkB_IkBn]<br>+ $k_{assoc\_NFkB\_IkB}$ * [NFkBc] * [IkBc]<br>- $k_{assoc\_IKK\_NFkBIkBc}$ * [IKK*] * [NFkB_IkBc]	export: NFkB-IkBn → NFkB-IkBc<br>association: NFkBc + IkBc → NFkB-IkBc<br>association: IKK* + NFkB-IkBc → NFkB-IKK-IkBc
$\dot{x}_{16}$	- $k_{export\_NFkBIkBn}$ * [NFkB_IkBn]<br>+ $k_{assoc\_NFKB\_IkB}$ * [NFkBn] * [IkBn]	export: NFkB-IkBn → NFkB-IkBc<br>association: NFkBn + IkBn → NFkB-IkBn
$\dot{x}_{17}$	$k_{assoc\_IKK\_IkBc}$ * [IKK*] * [IkBc]<br>- $k_{assoc\_IkBIKKc\_NFkBc}$ * [IKK_IkB] * [NFkBc]	association: IKK* + IkBc → IkB-IKKc<br>association: IkB-IKKc + NFkBc → NFkB-IkB-IKKc
$\dot{x}_{18}$	$k_{assoc\_IKK\_NFkBIkBc}$ * [IKK*] * [NFkB_IkBc]<br>+ $k_{assoc\_IkBIKKc\_NFkBc}$ * [NFkBc] * [IKK_IkB]<br>- $k_{deg\_NFkBIkBIKKc}$ * [NFkB_IKK_IkB]	association: NFkB-IkBc + IKK* → NFkB-IkB-IKKc<br>association: IkB-IKKc + NFkBc → NFkB-IkB-IKKc<br>degradation: NFkB-IkB-IKKc → IKK* + NFkBc
$\dot{x}_{19}$	- (1 - d_{IL10}) * $tau_{stabilize}$ * [Stabilizing_regulation] * 1 / (1 + tau_{IKKK} * max(0, [IKKK*] - $c_{stabilize}$))	time-dependent function
$\dot{x}_{20}$	1 - (1 - d_{IL10}) * (1 - 1 / (1 + exp(-(t - $tau_{destabilize}$))))	time-dependent function
$\dot{x}_{21}$	d_{LPS} * (k_{tx} * w_{NFkBtx_Tnf} * [NFkBn] / (1 + w_{NFkBtx_Tnf} * [NFkBn])) * (1 - dIL10 * (1 - (1 - max(t - tau_{IL10_1}, 0) / (tau_{IL10_2} - tau_{IL10_1})) * (1 - 1 / (1 + exp(-99 * (t - tau_{IL10_2})))))))<br>- $k_{deg\_mCherrym}$ * [mCherrym]	transcription: 0 → mCherrym; delayed effect of IL-10 pre-treatment<br>degradation: mCherrym → 0
$\dot{x}_{22}$	k_{tl} * [mCherrym]<br>- $k_{deg\_mCherry}$ * [mCherry]	translation: 0 → mCherry<br>degradation: mCherry → 0
$\dot{x}_{23}$	k_{mature} * [mCherry]<br>- $k_{deg\_mCherry}$ * [mCherryf]	maturation: mCherry → mCherryf<br>degradation: mCherryf → 0
$\dot{x}_{24}$	d_{LPS} * (k_{tx} * w_{NFkBtx_Tnf} * [NFkBn] / (1 + w_{NFkBtx_Tnf} * [NFkBn])) * (1 - d_{IL10} * (1 - (1 - max(t - tau_{IL10_1}, 0) / (tau_{IL10_2} - tau_{IL10_1})) * (1 - 1 / (1 + exp(-99 * (t - tau_{IL10_2})))))))<br>- k_{deg_Tnfm} * [Tnfm] * (1 / (1 + $w_{stabilize}$ * [Stabilizing_regulation]) + ($w_{maxdestabilize}$ - 1) * $w_{destabilize}$ * [Destabilizing_regulation] / (1 + $w_{destabilize}$ * [Destabilizing_regulation]))	transcription: 0 → TNFm; delayed effect of IL-10 pre-treatment<br>degradation: TNFm → 0
$\dot{x}_{25}$	k_{tl} * [Tnfm] * ((1 + $w_{destabilize}$ * [Destabilizing_regulation]) / (1 + $w_{maxdestabilize}$ * $w_{destabilize}$ * [Destabilizing_regulation]))<br>- $k_{secretion}$ * [TNF] * (1 - (1 + exp(-99 * (t - tau_{BFA})))^-1)<br>- k_{deg_TNF} * [TNF]	translation: 0 → TNF<br>secretion: TNF → TNFpool<br>degradation: TNF → 0
$\dot{x}_{EC1}$	(r1 / (1 + exp(-r_2 * (t - $tau_{density}$)))) * (1 - (1 + exp(-99 * (t - tau_{BFA})))^-1) * $k_{secretion}$ * SUM([TNF])<br>- $k_{deg\_TNFpool}$ * [TNFpool]	secretion: TNF → TNFpool<br>degradation: TNFpool → 0
$\dot{x}_{EC2}$	- k_{degLPS} * [LPS]	degradation: LPS → 0

Supplementary Notes

Supplementary Note 1

Below are the outcomes from four-way ANOVAs and Tukey's HSD tests. Null hypotheses were that there existed no effects of passaging, cell density at plating, conditioned media, treatment condition, or their pairwise interactions on the measured values.

Total EGFP-RelA signal in **Supplementary Fig. 2r** (a subset of the conditions are shown in **Fig. 2g**)

- Percentage of cells with high signal
  - Higher density passaging differed from standard ($p < 2 \times 10^{-16}$).
  - High density plating differed from low ($p = 1 \times 10^{-15}$).
  - High density media differed from low and fresh; low differed from fresh ($p < 2 \times 10^{-16}$).
  - LPS differed from no treatment ($p < 2 \times 10^{-16}$).
  - There was an interaction between passaging and density ($p = 0.034$).
  - There was an interaction between passaging and media ($p < 2 \times 10^{-3}$).
  - There was an interaction between density and media ($p = 8 \times 10^{-8}$).
  - There was an interaction between media and treatment ($p = 0.024$).

mCherry signal in **Supplementary Fig. 2r** (a subset of the conditions are shown in **Fig. 2g**)

- Percentage of cells with high signal
  - Higher density passaging differed from standard ($p < 2 \times 10^{-16}$).
  - High density plating differed from low ($p = 1 \times 10^{-10}$).
  - High density-conditioned media differed from low and fresh; low differed from fresh ($p = 8 \times 10^{-9}$).
  - LPS differed from no treatment ($p < 2 \times 10^{-16}$).
  - There was an interaction between passaging and treatment ($p = 1 \times 10^{-5}$).
  - There was an interaction between density and media ($p = 3 \times 10^{-3}$).

Supplementary Note 2

Below are the outcomes from two-way ANOVAs and Tukey's HSD tests. Null hypotheses were that there existed no effects of cell density, treatment condition, or their interaction on the measured values.

PE anti-TNF signal in **Fig. 5a**

- Letters refer to treatment conditions: V for BFA; W for sTNFR, LPS (1 ng ml^{-1}), and BFA; X for LPS (1 ng ml^{-1}) and BFA; Y for LPS (10 ng ml^{-1}) and BFA; and Z for LPS (100 ng ml^{-1}) and BFA.

- Mean signal
  - High density differed from 1/8 and 1/16; 1/4 differed from 1/16 ($p < 2 \times 10^{-16}$).
  - Treatment V differed from W, X, Y, and Z; W differed from Y and Z; X differed from Y and Z ($p = 8 \times 10^{-7}$).

- Percentage of cells with high signal
  - High density differed from 1/8 and 1/16; 1/4 differed from 1/8 and 1/16; 1/8 differed from 1/16 ($p < 2 \times 10^{-16}$).
  - Treatment V differed from W, X, Y, and Z ($p = 8 \times 10^{-12}$).
  - There was an interaction between density and treatment ($p = 2 \times 10^{-5}$).

Secreted factors in **Fig. 5b**

- Letters refer to treatment conditions: A for BFA; B for sTNFR, LPS (1 ng ml^{-1}), and BFA; C for LPS (1 ng ml^{-1}) and BFA; and D for LPS (100 ng ml^{-1}) and BFA.

- KC
    - High density differed from 1/4, 1/8, and 1/16 ($p = 2 \times 10^{-9}$).

- MCP-1
    - High density differed from 1/4, 1/8, and 1/16; 1/4 differed from 1/16 ($p = 4 \times 10^{-14}$).

- MIP-1α
    - High density differed from 1/4, 1/8, and 1/16 ($p = 5 \times 10^{-12}$).
    - Treatment A differed from B, C, and D; C differed from D ($p = 1 \times 10^{-6}$).
    - There was an interaction between density and treatment ($p = 5 \times 10^{-7}$).

- MIP-1β
    - High density differed from 1/4, 1/8, and 1/16; 1/4 differed from 1/16 ($p = 3 \times 10^{-10}$).
    - There was an interaction between density and treatment ($p = 0.026$).

- RANTES
    - High density differed from 1/4, 1/8, and 1/16; 1/16 differed from 1/4 and 1/8 ($p = 2 \times 10^{-13}$).

- TNF
    - High density differed from 1/4, 1/8, and 1/16 ($p = 3 \times 10^{-12}$).
    - Treatment D differed from A, B, and C ($p = 1 \times 10^{-8}$).
    - There was an interaction between density and treatment ($p = 3 \times 10^{-11}$).

PE anti-TNF signal in **Supplementary Fig. 5c**

- Letters refer to treatment conditions: J for no treatment; K for BFA; L for IL-10, LPS (1 ng ml^{-1}), and BFA; M for sTNFR, LPS (1 ng ml^{-1}), BFA; and N for LPS (1 ng ml^{-1}) and BFA.

- Mean signal
    - High density differed from 1/8 and 1/16; 1/4 differed from 1/8 and 1/16 ($p < 2 \times 10^{-16}$).
    - Treatment J differed from L, M, and N; K differed from L, M, and N; L differed from M and N ($p = 1 \times 10^{-7}$).
    - There was an interaction between density and treatment ($p = 6 \times 10^{-10}$).

- Percentage of cells with high signal
    - High density differed from 1/8 and 1/16; 1/4 differed from 1/8 and 1/16 ($p < 2 \times 10^{-16}$).
    - Treatment J differed from L, M, and N; K differed from L, M, and N; L differed from M and N ($p = 2 \times 10^{-15}$).
    - There was an interaction between density and treatment ($p < 2 \times 10^{-16}$).

Supplementary Methods

We developed a computational model to incorporate the new findings with prior knowledge on macrophage activation. We focused the scope to processes to which the new findings most directly pertain. This section describes prior knowledge and the model formulation, development, parameterization, and analysis. MATLAB files for homogeneous and heterogeneous models are provided as supplementary materials and detailed in **Supplementary Tables 1–4**.

Model formulation based upon prior knowledge

Cellular mechanisms and their representation in the model are described below. Salient state variables are in parentheses.

TLR4 signaling ($\dot{x}_1$, $\dot{x}_2$, $\dot{x}_5$, $\dot{x}_6$, $\dot{x}_{EC2}$)

Mechanism: LPS activates TLR4 signaling through two pathways named after the adaptor proteins MyD88 (Myeloid differentiation primary response gene 88) and TRIF (Toll/interleukin-1 receptor (TIR)-domain-containing adapter-inducing interferon-β)[1]. While these pathways differ in certain components and downstream effects, they overlap in NF-κB activation. MyD88 signaling involves the formation of a multi-subunit protein complex at the plasma membrane called the Myddosome, whose activity is induced and terminated rapidly. TRIF signaling requires TLR4 internalization to endosomes; signaling is initially delayed as activated TLR4 begins to reversibly shuttle from the plasma membrane to endosomes, but it is longer-lasting than MyD88 signaling. Signaling from activated receptors terminates after maturation of early endosomes to late endosomes.

Model: LPS has an initial value of 1 a.u., corresponding to a dose of 100 ng ml^{-1}. The inactive (TLR4) and active (TLR4*) forms of the receptor are assigned initial values of 0.1 and 0 a.u., respectively. Since LPS is in large molar excess of the receptor, its loss over time can be represented simply by first-order degradation. TLR4 is synthesized constitutively in its inactive form and undergoes first-order degradation. Receptor activation depends on LPS dose, not cell density. For the signaling cascade, we developed a reduced complexity version of a previously published model[1], e.g., that does not distinguish MyD88-mediated and TRIF-mediated signaling. As a general principle, we used a minimal number of variables to represent the most salient processes. During model development, we observed that reducing the granularity of certain mechanisms had little impact on the dynamics of interest such as NF-κB translocation.

NF-κB activation ($\dot{x}_7$, $\dot{x}_8$, $\dot{x}_{10}$, $\dot{x}_{11}$, $\dot{x}_{13}$, $\dot{x}_{14}$, $\dot{x}_{15}$, $\dot{x}_{16}$, $\dot{x}_{17}$, $\dot{x}_{18}$)

Mechanism: NF-κB is a dimer composed from five subunits: RelA (p65), cRel, RelB, p50, and p52[2]. In TLR4 signaling and in TNFR signaling, IKKK phosphorylates IKK (IκB kinase), which phosphorylates IκB, targeting IκB for degradation. The de-sequestered NF-κB can then translocate to the nucleus and induce target gene transcription. NF-κB is eventually re-sequestered by IκB, and NF-κB–IκB translocates to the cytoplasm. This activation and inactivation comprise one cycle of NF-κB nucleocytoplasmic translocation[1].

Model: we reduced and modified a portion of a previous model[1] while retaining NF-κB oscillatory behavior. Since RAW reporter cells express a functional EGFP-p65, and p65/p50 is the primary NF-κB dimer, we represent EGFP-p65-containing and native p65/p50 dimers as the same NF-κB variable. The variable IκB represents the IκBα gene product[3].

RelA feedback dominance switching ($\dot{x}_9$, $\dot{x}_{11}$)

Mechanism: RAW cells treated with LPS above a certain dose threshold enter a positive feedback loop in which NF-κB induces *Rela* expression[4]. This feedback dominance (FBD) switch counters the negative feedback from the induction of IκB by NF-κB. Since the switch requires *de novo* expression of the TF Ikaros, it takes effect starting several hours post-LPS. LPS at 100 ng ml^{-1} is well above the dose threshold.

Model: NF-κB undergoes the FBD switch in all cells at and above a presumed dose of 1 ng ml^{-1}. A time-dependent function was formulated for NF-κB-induced activity at the *Rela* promoter, based on previously published timecourse ChIP data[4] for NF-κB localization at the *Rela* promoter in RAW cells.

Early regulation of Tnf translation ($\dot{x}_{24}$, $\dot{x}_{25}$)

Mechanism: in the resting cell state, *Tnf* mRNA lacks a poly(A) tail and is not translated[5]. After LPS treatment, the mRNA is polyadenylated, allowing poly(A)-binding protein (PABP) to pseudo-circularize the mRNA, which increases ribosome recycling for rapid translation. TRIF signaling also promotes translation, by activating p38 mitogen activated protein kinase (MAPK), which activates MAP kinase-activated protein kinase 2 (MK2) to phosphorylate eukaryotic translation initiation factor 4E (eIF4E), which binds to the 5' mRNA cap and recruits the 40S ribosomal subunit. TRIF signaling also dephosphorylates eIF2, which de-represses translation by recruiting the 60S subunit[5].

Model: Since TNF production requires LPS treatment, the initial values of *Tnf* mRNA and TNF protein are set to zero regardless of the initial value of NF-κB.

Tnf post-transcriptional regulation ($\dot{x}_{19}$, $\dot{x}_{20}$, $\dot{x}_{24}$, $\dot{x}_{25}$)

Mechanism: Tnf mRNA is regulated post-transcriptionally through AU-rich elements (AREs) in its 5' UTR, with binding sites for over 20 proteins[5]. Some proteins such as Tristetraprolin (TTP) destabilize the mRNA by recruiting deadenylases and degradation factors, and others such as Hu-antigen R (HUR) stabilize the mRNA by competing with destabilizing proteins for occupancy[5]. In unstimulated macrophages, TTP is expressed at low levels[6]. Shortly after LPS treatment, kinases including p38 and Erk are activated and *Tnf* mRNA is stabilized[7]. However, TLR4 and TNFR signaling also induce TTP expression via p38 and ERK signaling[8] (and IL-10R signaling also induces TTP expression, via STAT3[6]). TTP binds to *Tnf* mRNA and leads to its destabilization[9]. The outcome is a limited-duration burst in TNF expression[7].

Model: To capture the limited-duration burst in TNF expression while limiting model complexity, stabilizing regulation (SR) is represented by one variable and destabilizing regulation (DSR) is represented by another. SR becomes active after LPS (downstream of TLR4* and TNFR* via IKKK*), and it decreases in activity over time. SR slows the degradation of *Tnf* mRNA. DSR becomes active after a delay. DSR increases *Tnf* mRNA degradation and suppresses TNF translation.

Action of IL-10 pre-treatment through STAT3 (represented through a parameter, not state variables)

Mechanism: Bcl-3 is a nuclear-localized protein that dimerizes with p50 or p52 and binds NF-κB-responsive promoters[10]. Bcl-3 expression is induced by STAT3 in IL-10R signaling.

Model: To limit model complexity, rather than introducing variables for IL-10R signaling, STAT3 activation, or Bcl-3 interactions, we found that representing the effect of IL-10 on FBD via a fitted parameter was sufficient to capture the observed decrease in reporter expression (**Fig. 2a**).

Action of IL-10 pre-treatment through MAPKs ($\dot{x}_{19}$, $\dot{x}_{20}$)

Mechanism: MAPKs regulate the initial response to LPS and the resolution[11]. Their effects on downstream targets are complex and have been described as *incoherent*—having seemingly opposing effects. Signaling via MyD88 and TRIF activates p38, extracellular-signal-regulated kinases 1 and 2 (ERK1/2), and c-Jun N terminal kinase (JNK). MK2, a phosphorylation target of p38, regulates *Tnf* and *Il10* mRNA stability by: (a) preventing recruitment of the adenylase CCR4-associated factor 1 (CAF1), thereby preventing proteins like TTP from destabilizing target mRNAs, and (b) inducing TTP expression. IL-10R signaling also regulates *Tnf*, by activating STAT3 (which induces TTP) and increasing expression of dual specific phosphatase 1 (DUSP1, which dephosphorylates p38 and inhibits late-phase p38 activity)[11]. In macrophages, IL-10R signaling destabilizes inflammatory cytokine mRNAs like *Tnf* that contain 3' UTR AU-rich elements (AREs), by: (a) repressing LPS-induced activation of p38 MAPK, and (b) inhibiting expression of HuR, a protein that stabilizes mRNAs by binding AREs[12].

Model: IL-10 treatment decreases stabilizing regulation and increase destabilizing regulation of *Tnf* mRNA. Effects of the MAPKs are represented by SR downstream of IKKK activation, and effects of TTP are represented by DSR. IL-10 pre-treatment prevents SR, and it activates DSR by 0 hps.

TNFR activation ($\dot{x}_3$, $\dot{x}_4$, $\dot{x}_5$, $\dot{x}_6$, $\dot{x}_{EC1}$)

Mechanism: Extracellular TNF binds TNFR1, and the receptor-ligand complex is internalized[13]. Adaptor proteins activate IKKK and MAPK signaling, leading to NF-kB activation.

Model: the TNF pool has an initial value of 0 a.u. The inactive (TNFR) and active (TNFR*) forms of the receptor are assigned initial values of 0.1 and 0 a.u., respectively, analogous to the TLR4 receptor. TNFR* and TLR4* converge at IKKK activation, and thus overlap in regulating *Tnf*.

Cell density ($\dot{x}_{EC1}$)

Mechanism: Cell density affects the proportion of highly activated cells and the amount of secreted TNF.

Model: To account for population growth over time, the rate at which secreted TNF contributes to the extracellular TNF pool is multiplied by a time-dependent function. The multiplier is applied to the pool, not individual cells. The proportion of highly activated cells is based on experimental observations.

Blockade of TNFR signaling ($\dot{x}_3$, $\dot{x}_4$)

Mechanism: At 1 h pre-LPS, cells were treated with soluble TNF receptor (sTNFR), which competes with surface TNFR for TNF. The dose of sTNFR was chosen based on a prior study such that it would be in molar excess of secreted TNF[14].

Model: sTNFR blockade prevents TNFR activation. As a result, following LPS treatment, downstream nodes such as IKKK are activated to a lesser extent than they would be with paracrine feedback.

Secretion ($\dot{x}_{25}$, $\dot{x}_{EC1}$)

Mechanism: p38 modulates the activity of proteins involved in endocytic trafficking to enhance cytokine secretion during the LPS response. At the cell surface, TNF precursor is released in soluble form following cleavage by TNFα-converting enzyme (TACE), which is activated by an LPS-activated lipid hydrolase[11].

Model: since simulations begin at 0 hps, TNF secretion is assigned a constant rate parameter.

Blockade of secretion ($\dot{x}_{25}$, $\dot{x}_{EC1}$)

Mechanism: Brefeldin A (BFA) prevents secretion involving Golgi transport. After BFA treatment, TNF is no longer secreted, and it can accumulate intracellularly.

Model: A time-dependent step-down function represents prevention of TNF secretion. Since some TNF is secreted prior to BFA, some paracrine signaling occurs, though to a lesser extent than without BFA.

Model formulation for other cellular processes

Receptor activation

Receptors are synthesized constitutively in an inactive form and are initially at steady state. Receptors become activated through a second-order reaction with the extracellular cue, and initiate the downstream cascade. Certain rate constants differ for TLR4 and TNFR.

Kinase cascade

Kinases are present at a constant level. They are initially inactive, and become activated by a second-order reaction with an upstream node. Basal deactivation is first-order. For example, TLR4* and IKKK react to convert IKKK to IKKK*, which eventually returns to IKKK (due to the action of phosphatases).

Translocation

Translocation between the cytoplasm and nucleus is first order. Rate constants differ by species (NF-κB, IκB, and NF-κB–IκB) and direction of movement.

Transcription

Transcription was formulated using fractional activation. Terms for transcriptional activators are in both the numerator and denominator. Terms for inhibitory effects are in the denominator.

Translation

Translation is generally treated as first order with mRNA. However, *Tnf* translation has additional regulation.

Secretion

There are 30 cells in the model, and the sum of their TNF secretion contributes to the extracellular pool.

Degradation

Degradation is generally treated as first order. Cases involving regulated degradation are nonlinear.

Model development, parameterization, and implementation

Before parameterizing the full model, we started with a model of cell-intrinsic effects on NF-κB that includes TLR4 signaling, regulation of NF-κB activation, and RelA and IκB expression (**Supplementary Fig. 3a**). Following LPS treatment, TLR4 is activated to TLR4*, which activates IKKK to IKKK*, which activates IKK to IKK*. In the cytoplasm, IKK* associates with IκB or NF-κB–IκB to form IKK–IκB or NF-κB–IKK–IκB, respectively. IKK–IκB and NF-κB also associate to form NF-κB–IKK–IκB, and IKK targets IκB for degradation. (Formally, degradation occurs regardless of whether NF-κB is complexed, but for the purpose of model reduction we represented this process for NF-κB–IKK–IκB only. This decision did not have a noticeable impact on NF-κB activity.) The reaction releases NF-κB and IKK, and NF-κB can then enter the nucleus and induce transcription of *Rela* and *Ikba*. NF-κB, IκB, and NF-κB–IκB translocate between the nucleus and cytoplasm. Since NF-κB–IκB exits the nucleus with a much faster rate constant than that with which it enters, its translocation is treated as unidirectional. As with the representation of the effect of IKK on IκB, this model reduction had no discernable impact. During model development, we encountered many such instances where complexity could be reduced for reasons such as separation of timescales, redundant pathway effects, or negligible reaction fluxes.

To conduct an broad search of parameter space for fitting the model, we tested many parameter sets using a Sobol sequence—a pseudorandom number list that uniformly samples the unit hypercube in the limit of the sequence[15]. This initial sweep was followed by multi-objective optimization using a genetic algorithm with many generations, each comprising the following steps:

1. Evaluation: quantify goodness of fit for each parameter set based on the deviation of simulated outcomes from experimental data.
2. Selection: identify parameter sets that yield the best fits in the current generation. Stringent criteria were applied to eliminate sets for which the population-mean simulated outcomes fell outside of any specified windows of acceptable deviation from the population-mean experimental data.
3. Repopulation: replicate the selected sets to restore the population size.
4. Mutation: introduce random variation to the parameter values, drawing from Gaussian distributions centered on current values. Simulated annealing was used, in which coefficients of variation were decreased after many generations, to narrow in on solutions.

The algorithm yielded a family of similar-performing four-parameter sets for the NF-κB module. These sets were carried forward and sampled during the fitting of the remaining eight parameters in the full model as described for **Fig. 3**, and this fit yielded the homogeneous (one-cell) model. Fitting, simulations, and analysis were conducted in MATLAB.

To investigate the NF-κB module independent of TNF intercellular feedback, the homogeneous model was run under various conditions (**Supplementary Fig. 3b**). The results depict how, following TLR4 activation, NF-κB enters the nucleus and induces target gene transcription. Damped oscillations are attributable to the negative feedback between NF-κB activation and IκB expression and the time required for *de novo* IκB expression. We find that depending on the LPS dose, FBD switch, and initial value of inactive NF-κB, simulations yield qualitatively distinct trajectories for total and nuclear NF-κB, consistent with wide-ranging outcomes observed by confocal microscopy (**Fig. 2, Supplementary Fig. 2**).

A population of 30 cells was generated using equivalent intracellular state variables and reactions, which all had access to LPS and the extracellular TNF pool. Heterogeneity was introduced by assigning differences to the basal transcription rate of NF-κB RNA, initial value of NF-κB RNA, and initial value of cytoplasmic NF-κB–IκB (corresponding to 0 hps). These values are proportional to confocal microscopy measurements for the 30 cells at high density. Values for cells at low density, in equivalent units, were obtained as described for **Fig. 3b**.

Model parameterization: cell growth

RAW cell density was monitored over time for different cell densities and treatment conditions. These data were used to fit a logistic model for time-dependent growth (**Supplementary Fig. 3c**).

Model formulation: representing the experimental perturbations

Perturbations were modeled as described below and depicted in **Supplementary Fig. 3d**. Parameters are denoted by k for kinetic processes and w for weights in transcriptional regulation. Variables are abbreviated as: NFkBn, nuclear NF-κB; Tnfm, *Tnf* mRNA; TNF, intracellular TNF; TNFpool, extracellular TNF pool; SR, stabilizing regulation on *Tnf* mRNA; DSR, destabilizing regulation on *Tnf* mRNA.

Terms containing δ indicate a treatment or perturbation that is either present or absent for each scenario to be analyzed.

$$\delta_{\mathrm{IL10}} = \begin{cases} no\ \mathrm{IL10} \longrightarrow 0 \\ \mathrm{IL10} \longrightarrow 1 \end{cases} \tag{1}$$

$$\delta_{\mathrm{sTNFR}} = \begin{cases} no\ \mathrm{sTNFR} \longrightarrow 0 \\ \mathrm{sTNFR} \longrightarrow 1 \end{cases} \tag{2}$$

$$\delta_{\mathrm{LPS}} = \begin{cases} no\ \mathrm{LPS} \longrightarrow 0 \\ \mathrm{LPS} \longrightarrow 1 \end{cases} \tag{3}$$

$$\delta_{\mathrm{BFA}} = \begin{cases} no\ \mathrm{BFA} \longrightarrow 0 \\ \mathrm{BFA} \longrightarrow 1 \end{cases} \tag{4}$$

Time-dependent cell density (ρ) is described by a logistic equation. Units are relative to high cell density (1 a.u.) at the time of LPS treatment ($t = 0$). From a fit to data (**Supplementary Fig. 3c**) with constraints for eight-fold difference at plating and for equal density in the limit of time, the horizontal asymptote for maximum density (r_1) is 1.99 a.u., the rate of logistic growth (r_2) is 0.0365 h^{-1}, and τ_{density} is –0.247 h for high density and 62.6 h for low density.

$$\rho\left(t, \tau_{\mathrm{density}}\right) = \frac{r_1}{1 + \mathrm{e}^{-r_2 \cdot (t - \tau_{\mathrm{density}})}} \tag{5}$$

Total TNF secretion by cells i=1:N, accounting for population growth and sTNFR pre-treatment, is:

$$\rho \cdot (1 - \delta_{\mathrm{sTNFR}}) \cdot k_{\mathrm{secretion}} \cdot \sum_{i}^{N} [\mathrm{TNF}_i] \tag{6}$$

The effect of FBD on *Rela* transcription is represented by a unitless time-dependent function $\mathrm{F_{FBD}}$, which was formulated and fitted based on timecourse ChIP data[4] for RelA localization at the *Rela* promoter post-LPS.

$$\mathrm{F_{FBD}} = \frac{1}{6} \cdot \delta_{\mathrm{LPS}} \cdot t^3 \cdot e^{-t} \tag{7}$$

Rela transcription involves basal transcription, inducible transcription including the effect of FBD, and the inhibitory effect of IL-10 pre-treatment.

$$k_{\mathrm{tx}} \frac{w_{\mathrm{basaltxNFkB}} + \mathrm{F_{FBD}} \cdot w_{\mathrm{NFkBtxNFkB}} \cdot [\mathrm{NFkBn}]}{1 + \mathrm{F_{FBD}} \cdot w_{\mathrm{NFkBtxNFkB}} \cdot ([\mathrm{NFkBn}] + w_{\mathrm{IL10txFBD}} \cdot \delta_{\mathrm{IL10}})} \tag{8}$$

Tnf and *mCherry* transcription are each induced by nuclear NF-κB.

$$F_{\mathrm{NFkB\_Tnf}} = w_{\mathrm{NFkBtxTnf}} \cdot [\mathrm{NFkBn}] \quad (9)$$

An additional consideration with IL-10 pre-treatment is that we experimentally observed a decrease in *Tnf* promoter activity beginning after some delay post-LPS. This can be described by a time-dependent ramp down function ($F_{\mathrm{IL10\_Tnf}}$) starting at time $\tau_{\mathrm{IL10\_1}}$ and ending at time $\tau_{\mathrm{IL10\_2}}$, where H is the Heaviside function.

$$F_{\mathrm{IL10\_Tnf}} = 1 - \delta_{\mathrm{IL10}} \cdot \left(1 - \frac{\max\left(t - \tau_{\mathrm{IL10\_1}}, 0\right)}{\tau_{\mathrm{IL10\_2}} - \tau_{\mathrm{IL10\_1}}}\right) \cdot \left(1 - \frac{1}{1 + H\left(t - \tau_{\mathrm{IL10\_2}}\right)}\right) \quad (10)$$

Transcription from the *Tnf* promoter, incorporating IL-10's effect, is:

$$\delta_{\mathrm{LPS}} \cdot k_{\mathrm{tx}} \cdot \frac{F_{\mathrm{NFkB\_Tnf}}}{1 + F_{\mathrm{NFkB\_Tnf}}} \cdot F_{\mathrm{IL10\_Tnf}} \quad (11)$$

For post-transcriptional regulation of *Tnf* mRNA, there are two opposing effects: stabilizing regulation slows the degradation, and destabilizing regulation promotes the degradation.

$$F_{\mathrm{stabilize}} = \frac{1}{1 + w_{\mathrm{stabilize}} \cdot [\mathrm{SR}]} \quad (12)$$

$$F_{\mathrm{destabilize}} = \frac{w_{\mathrm{destabilize}} \cdot [\mathrm{DSR}] \cdot (w_{\mathrm{maxdestabilize}} - 1)}{1 + w_{\mathrm{destabilize}} \cdot [\mathrm{DSR}]} \quad (13)$$

With these effects, *Tnf* mRNA degradation is:

$$k_{\mathrm{degTnfm}} \cdot [\mathrm{Tnfm}] \cdot (F_{\mathrm{stabilize}} + F_{\mathrm{destabilize}}) \quad (14)$$

Destabilizing regulation also acts to repress translation of the TNF protein:

$$F_{\mathrm{repress\_tl}} = \frac{1 + w_{\mathrm{destabilize}} \cdot [\mathrm{DSR}]}{1 + w_{\mathrm{maxdestabilize}} \cdot w_{\mathrm{destabilize}} \cdot [\mathrm{DSR}]} \quad (15)$$

Translational repression is represented as:

$$k_{\mathrm{tl}} \cdot [\mathrm{Tnfm}] \cdot F_{\mathrm{repress\_tl}} \quad (16)$$

sTNFR pre-treatment blocks TNFR signaling. Its effect is a zero multiplier on TNFR activation.

$$(1 - \delta_{\mathrm{sTNFR}}) \cdot k_{\mathrm{activateTNFR}} \cdot [\mathrm{TNFR}] \cdot [\mathrm{TNFpool}] \quad (17)$$

BFA prevents secretion starting at the time of BFA treatment (τ_{BFA}).

$$\delta_{\mathrm{BFA}}(t, \tau_{\mathrm{BFA}}) = 1 - H(t - \tau_{\mathrm{BFA}}) \quad (18)$$

BFA's effect is represented by a step-down multiplier on TNF secretion for each cell.

$$k_{\mathrm{secretion}} \cdot \delta_{\mathrm{BFA}} \cdot [\mathrm{TNF}] \quad (19)$$

State variables, parameters[1,4,16-19], stimulus-specific and perturbation-specific parameters, and ODEs are provided in **Supplementary Tables 1–4**.

Supplementary References

1 Cheng, Z., Taylor, B., Ourthiague, D. R. & Hoffmann, A. Distinct single-cell signaling characteristics are conferred by the MyD88 and TRIF pathways during TLR4 activation. *Sci Signal* **8**, ra69 (2015).

2 Basak, S., Behar, M. & Hoffmann, A. Lessons from mathematically modeling the NF-κB pathway. *Immunol Rev* **246**, 221–238 (2012).

3 Keogh, B. & Parker, A. E. Toll-like receptors as targets for immune disorders. *Trends Pharm Sci* **32**, 435–443 (2011).

4 Sung, M.-H. *et al.* Switching of the relative dominance between feedback mechanisms in lipopolysaccharide-induced NF- κB signaling. *Sci Signal* **7**, ra6 (2014).

5 Carpenter, S., Ricci, E. P., Mercier, B. C., Moore, M. J. & Fitzgerland, K. A. Post-transcriptional regulation of gene expression in innate immunity. *Nat Rev Immunol* **14**, 361–376 (2014).

6 Gaba, A. *et al.* IL-10−mediated tristetraprolin induction is part of a feedback loop that controls macrophage STAT3 activation and cytokine production. *J Immunol* **189**, 2089–2093 (2012).

7 Gais, P. *et al.* TRIF signaling stimulates translation of TNF-α mRNA via prolonged activation of MK2. *J Immunol* **184**, 5842–5848 (2010).

8 Kafasla, P., Skliris, A. & Kontoyiannis, D. L. Post-transcriptional coordination of immunological responses by RNA-binding proteins. *Nat Immunol* **15**, 492–502 (2014).

9 Brooks, S. A. & Blackshear, P. J. Tristetraprolin (TTP): interactions with mRNA and proteins, and current thoughts on mechanisms of action. *Biochim Biophys Acta* **1829**, 666–679 (2013).

10 Bonizzi, G. & Karin, M. The two NF-κB activation pathways and their role in innate and adaptive immunity. *Trends Immunol* **25**, 280–288 (2004).

11 Bode, J. G., Ehlting, C. & Häussinger, D. The macrophage response towards LPS and its control through the p38MAPK–STAT3 axis. *Cell Signal* **24**, 1185–1194 (2012).

12 Rajasingh, J. *et al.* IL-10-induced TNF-alpha mRNA destabilization is mediated via IL-10 suppression of p38 MAP kinase activation and inhibition of HuR expression. *FASEB J* **20**, E1393–E1403 (2006).

13 Parameswaran, N. & Patial, S. Tumor necrosis factor-α signaling in macrophages. *Crit Rev Eukaryot Gene Expr* **20**, 87–103 (2010).

14 Covert, M. W., Leung, T. H., Gaston, J. E. & Baltimore, D. Achieving stability of lipopolysaccharide-induced NF-κB activation. *Science* **309**, 1854–1857 (2005).

15 Sobol, I. M. Uniformly distributed sequences with an additional uniform property. *USSR Comp Math Math+* **16**, 1332–1337 (1976).

16 Werner, S. L., Barken, D. & Hoffmann, A. Stimulus specificity of gene expression programs determined by temporal control of IKK activity. *Science* **309**, 1857–1861 (2005).

17 Caldwell, A. B., Cheng, Z., Vargas, J. D., Birnbaum, H. A. & Hoffmann, A. Network dynamics determine the autocrine and paracrine signaling functions of TNF. *Genes Dev* **28**, 2120–2133 (2014).

18 Kalita, M. K. *et al.* Sources of cell-to-cell variability in canonical Nuclear Factor-κB (NF-κB) signaling pathway inferred from single cell dynamic images. *J Biol Chem* **286**, 37741–37757 (2011).

19 Maiti, S., Dai, W., Alaniz, R. C., Hahn, J. & Jayaraman, A. Mathematical modeling of pro- and anti-inflammatory signaling in macrophages. *Processes* **3**, 1–18 (2015).
