# Supplementary material for: Macrophages employ quorum licensing to regulate collective activation
Source: Nat Commun. 2020 Feb 13;11:878. doi: 10.1038/s41467-020-14547-y (PMC7018708; doi:10.1038/s41467-020-14547-y)
Supplement: Supplementary file 3 — Description of Additional Supplementary Files [file 41467_2020_14547_MOESM3_ESM.pdf]

Description of Additional Supplementary Files

File Name:Supplementary Software 1
Description: This archive contains model files
