# Supplementary material for: Macrophages employ quorum licensing to regulate collective activation
Source: Nat Commun. 2020 Feb 13;11:878. doi: 10.1038/s41467-020-14547-y (PMC7018708; doi:10.1038/s41467-020-14547-y)
Supplement: Supplementary file 4 — Supplementary Software 1 [file 41467_2020_14547_MOESM4_ESM.zip › Supplementary Software 1/Description of code files.rtf]

Code files1. Macrophage_model_homogeneous.mOne-cell model.2. Macrophage_model_heterogeneous.mPopulation model.3. sim_cell.matExample output from a homogeneous simulation.4. sim_population.matExample output from a heterogeneous simulation.
