# Supplementary material for: Macrophages employ quorum licensing to regulate collective activation
Source: Nat Commun. 2020 Feb 13;11:878. doi: 10.1038/s41467-020-14547-y (PMC7018708; doi:10.1038/s41467-020-14547-y)
Supplement: Supplementary file 4 — Supplementary Software 1 [file 41467_2020_14547_MOESM4_ESM.zip › Supplementary Software 1/README.rtf]

Macrophages employ quorum licensing to regulate collective activationDOI 10.5281/zenodo.35379421. System requirementsThe code can be run using MATLAB which can be obtained from Mathworks at https://www.mathworks.com/products/matlab.html.Code was developed and tested on macOS Sierra.2. Installation guideNo specific installation is required other than for MATLAB.3. DemoFiles can be run in MATLAB, and they will produce an output argument containing the simulated outcomes. The expected runtime is < 1 second for the homogeneous model and < 1 minute for the heterogeneous model on a standard desktop computer.4. Instructions for useSimulation conditions can be adjusted in the upper section of each file.
