# Supplementary material for: Macrophages employ quorum licensing to regulate collective activation
Source: Nat Commun. 2020 Feb 13;11:878. doi: 10.1038/s41467-020-14547-y (PMC7018708; doi:10.1038/s41467-020-14547-y)
Supplement: Supplementary file 5 — Source Data [file 41467_2020_14547_MOESM5_ESM.zip › Source Data/Confocal Microscopy/Description of confocal microscopy files.rtf]

Confocal microscopy filesThis file lists data matrices, corresponding figures, descriptions, and dimensions for confocal microscopy. Files can be opened in MATLAB.HD_LPSFig. 2fHigh density with LPS treatment; mCherry22 rows for time points 2 to 23 h30 columns for cellsIL10_LPSFig. 2fHigh density with IL-10 pre-treatment and LPS treatment; mCherry24 rows for time points 0 to 23 h30 columns for cellsLD_EGFPRelAFig. 2f, Supplementary Fig. S2kLow density with LPS treatment; EGFP-RelA20 vectors (one per cell), with the first row for the time point and the second row for the valueLD_mCherryFig. 2f, Supplementary Fig. S2kLow density with LPS treatment; mCherry20 vectors (one er cell), with the first row for the time point and the second row for the valueHD_sTNFR_LPSFig. 2b–f, Supplementary Fig. S2l–oHigh density with sTNFR pre-treatment and LPS treatmentFour matrices for nuclear, cytoplasmic, and total EGFP-RelA, and mCherry28 columns for time points 0 to 27 h30 rows for cells
