# Supplementary material for: Macrophages employ quorum licensing to regulate collective activation
Source: Nat Commun. 2020 Feb 13;11:878. doi: 10.1038/s41467-020-14547-y (PMC7018708; doi:10.1038/s41467-020-14547-y)
Supplement: Supplementary file 5 — Source Data [file 41467_2020_14547_MOESM5_ESM.zip › Source Data/Flow Cytometry/Description of flow cytometry files.rtf]

Flow cytometry filesThis file lists data structures and matrices, corresponding figures, descriptions, and dimensions for flow cytometry. Files can be opened in MATLAB.RAW cells: TNF intracellular stainFor TNF data structures:IC: PE isotope control antibodyab: PE anti-TNF antibodyfig1b_IL10Fig. 1b, Supplementary Fig. S1aL: 100 ng/ml LPSI2_L: 2 ng/ml IL-10, 100 ng/ml LPSI10_L: 10 ng/ml IL-10, 100 ng/ml LPSfig1c_BFAFig. 1c, Supplementary Fig. S1cL: LPS treatmentIL: IL-10 pre-treatment, LPS treatmentI: IL-10 pre-treatmentnt: no ligand treatment3b0: 3 h without BFA2b1: 2 h without and 1 h with BFA1b2: 1 h without and 2 h with BFAfig1d_densityFig. 1d, Supplementary Fig. S1dL: LPS treatmentIL: IL-10 pre-treatment, LPS treatmentI: IL-10 pre-treatmentnt: no ligand1: high density2: one-half density8: one-eighth density3: 3 hps6: 6 hps12: 12 hpsfig1e_sTNFRFig. 1e, Supplementary Fig. S1eHD: high densityLD: low densitynt: no treatmentLPS: LPS treatmentblockade: sTNFR pre-treatmentReporter cells: total EGFP-RelA and mCherryfig2a_reportersFig. 2a, Supplementary Fig. S2b–cnt: no treatmentLPS: LPS treatment0: 0 hps12: 12 hps1: high density2: low density3: high density, IL-10 pre-treatment4: high density, sTNFR pre-treatmentfig2g_standard, fig2g_higherFig. 2g, Supplementary Fig. S2rHD: high densityLD: low densityHM: high density-conditioned mediaLM: low density-conditioned mediaFM: fresh mediant: no treatment LPS: LPS treatment1: replicate 12: replicate 23: replicate 3fig4a_dosesFig. 4a, Supplementary Fig. S4aHD: high densityLD: low densityVLD: very low densitynt: no treatmentLPS: LPS treatmentPMA: PMA treatment0: none01: 0.1 ng/ml1: 1 ng/ml10: 10 ng/ml100: 100 ng/ml1: replicate 12: replicate 23: replicate 3BMM: TNF intracellular stain and secreted analytesFor TNF data structures:IC: PE isotope control antibodyTNF: PE anti-TNF antibodyfig5aFig. 5aG1: BFAG2: sTNFR, LPS (1 ng/ml), BFAG3: LPS (1 ng/ml), BFAG4: LPS (10 ng/ml), BFAG5: LPS (100 ng/ml), BFAD1: one-sixteenth densityD2: one-eighth densityD3: one-fourth densityD4: high densityR1: replicate 1R2: replicate 2R3: replicate 3figS5cSupplementary Fig. S5cG1: no treatmentG2: BFAG3: IL-10, LPS (1 ng/ml), BFAG4: sTNFR, LPS (1 ng/ml), BFAG5: LPS (1 ng/ml), BFAD1: one-sixteenth densityD2: one-eighth densityD3: one-fourth densityD4: high densityR1: replicate 1R2: replicate 2R3: replicate 3figS5dFig. 5b, Supplementary Fig. S5d–fFields are the mean and S.E.M. for the analytes, numbered 1–23: IL-1alpha, IL-1beta, IL-2, IL-3, IL-4, IL-5, IL-6, IL-9, IL-10, IL-12p40, IL-12p70, IL-13, IL-17, Eotaxin, G-CSF, GM-CSF, IFN-gamma, KC, MCP-1, MIP-1alpha, MIP-1beta, RANTES, TNF.
